# Zuun Baruun Kherem, a medieval Eurasian center in Eastern Mongolia

**DOI:** 10.1007/s41826-025-00113-2

**Published:** 2025-10-07

**Authors:** Joshua Wright, Lance Pursey, Sarah Pleuger-Dreibrodt, Anna Misterkiewicz, Batdalai Byambatsuren, Emilie Jean Green

**Affiliations:** 1 Department of Archaeology, University of Aberdeen, St Mary’s, Elphinstone Road, Aberdeen, AB24 3UF UK; 2 Faculty of Letters, Arts and Sciences, Waseda University, Tokyo, Japan; 3 Department of Asian Studies, The Hebrew University, Mt. Scopus, Jerusalem, 91905 Israel; 4 School of Classics and Archaeology, Old Medical School, Elsie Inglis Quadrangle, University of Edinburgh, Teviot Place, Edinburgh, EH8 9AG UK; 5 Department of Anthropology and Archaeology, National University of Mongolia, P.O. Box - 330, University Building 2, Room 202, Baga toiruu- 47, Ulaanbaatar 46-a, Sukhbaatar duureg, 14201 Mongolia

**Keywords:** Kitan-Liao, Urban, Medieval, Fauna, Chronology

## Abstract

Mongolia contains many urban sites of the medieval Kitan-Liao period (10th-12th centuries CE). The paired site of Zuun Baruun Kherem (ZBK) is a well-preserved example. The ZBK project aimed to examine the form and chronology of construction, the empty areas contained within many walled enclosures of this period, and the administrative use of these centers on the imperial frontier. Fieldwork was carried out primarily using hand augers to systematically examine subsurface deposits and a photogrammetric digital elevation model for detailed study of the surface topography. As well as ceramics, a surprising amount of datable bone and charcoal was recovered. Most remains focused on the densely inhabited Zuun Kherem. Baruun Kherem appears to be a mostly empty space with a few large elite structures inside. The enclosures were in use between the early 10th century and the early 12th century CE. Extensive midden deposits in Zuun Kherem illustrate urban animal penning as well as a distinctive set of ceramic vessels. Inhabitation of Baruun Kherem appears to be more akin to mobile habitation of non-urban settings.

**Supplementary Information:**

The online version contains supplementary material available at 10.1007/s41826-025-00113-2.

## Introduction

**Fig. 1:**
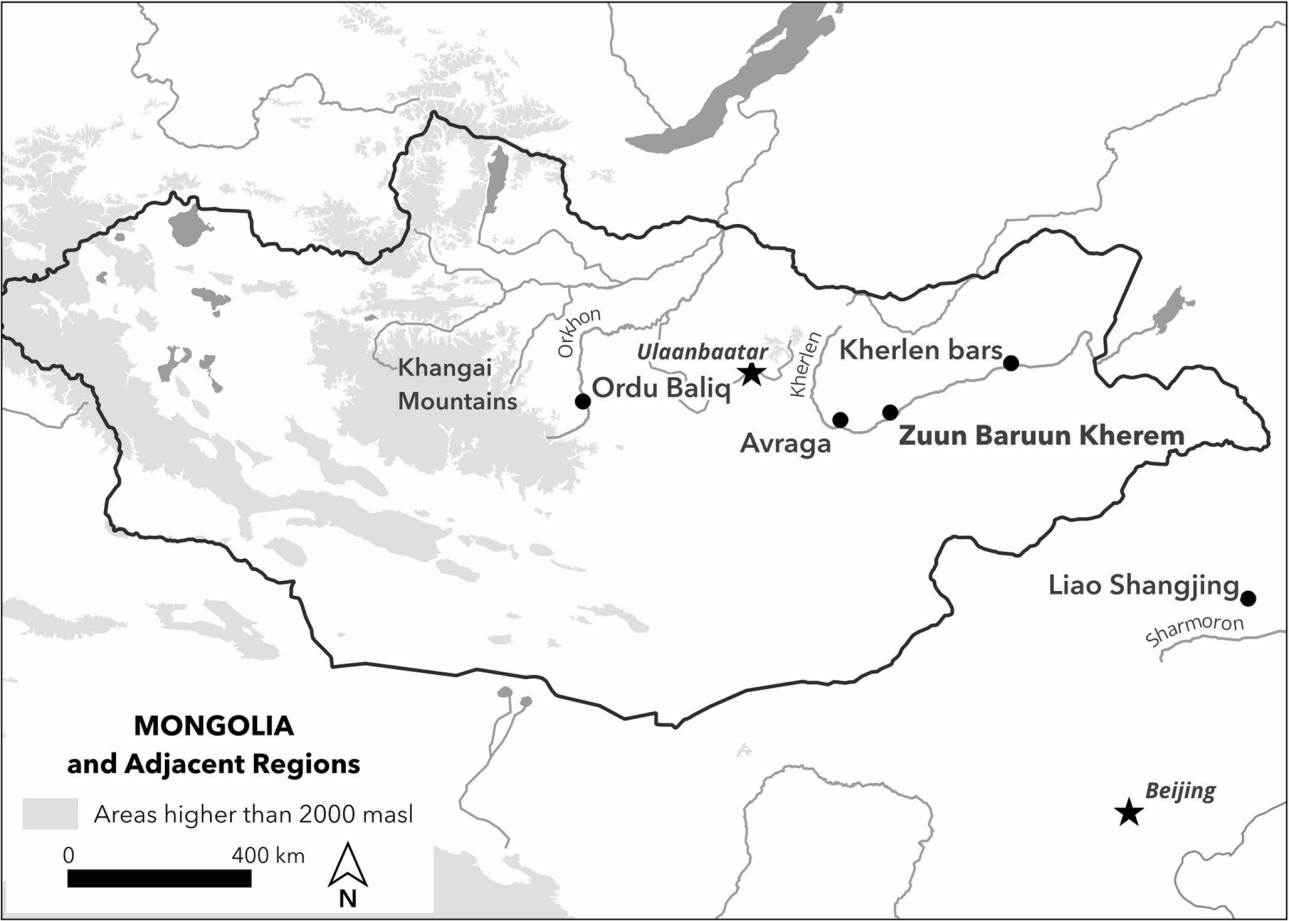
Mongolia and adjacent regions showing sites and rivers mentioned here

In the last years of the 10th century CE the Kitan rulers of the Liao dynasty (916–1125 CE) extended their power into what is now central Mongolia. Securing territory as far west as the Khangai highlands and the Upper Orkhon watershed (Fig. 1). After the initial consolidation they directed towns to be built or refurbished and garrisoned the country. By the first half of the twelfth century these towns were being transformed or abandoned as the Liao empire collapsed and new powers filled the void. The period of almost four centuries between the fall of the Uyghur Khanate in 848 CE and the rise of the Mongol Empire in the early 13th century CE has been called a “blank period” (Shiraishi 2001) or period of “anarchy” (Barfield 1989) in the history of the Mongolian Steppe. “Blank” due to the dearth of written sources, and “anarchy” due to the absence of control by any one state or nomadic empire. With this as a backdrop, archaeology is able to fill in the long “Kitan Century” in Mongolia giving life and order to a period where the region was not at the heart of an empire, but at its dynamic periphery.

The Kitan Liao State came into existence during a geopolitical realignment in East Asia.

Prior to the tenth century CE the key power on the Mongolian Plateau was the Uyghur Khanate (744–848 CE) based in the Orkhon river basin. This empire was destroyed by an invasion from the north, when Kirghiz sacked the Uyghur capital Ordu Baliq. Beginning in early tenth century, the first emperor of the Kitan Liao empire Abaoji (r.916–926 CE) and his successor, Deguang (r.927–947) consolidated control of the Sharmoron basin and began to campaign not only in the central plains and loess plateau regions to the south, but also to their northeast and northwest, bringing the Manchurian Plain and Mongolian Plateau into their sphere of control. In the tenth century these rulers also initiated an unprecedented transfer of populations from the North Chinese and the Manchurian Plain into the new imperial core of the Kitan Liao in the Sharmoron basin.

Construction of walled sites like Zuun and Baruun Kherem seem to begin after a shift in policy towards the steppe starting in 994 CE, when the Liao court took a more expansionist and interventionist stance on the Mongolian Plateau. Prior to this the official Liao policy was to pit the interests of various groups in Mongolia against each other to keep them in line, and to relocate some groups closer to the Liao heartlands. Intermittent peace and warfare continued throughout the 11th century, until the withdrawal of Liao forces from the region.

**Fig. 2:**
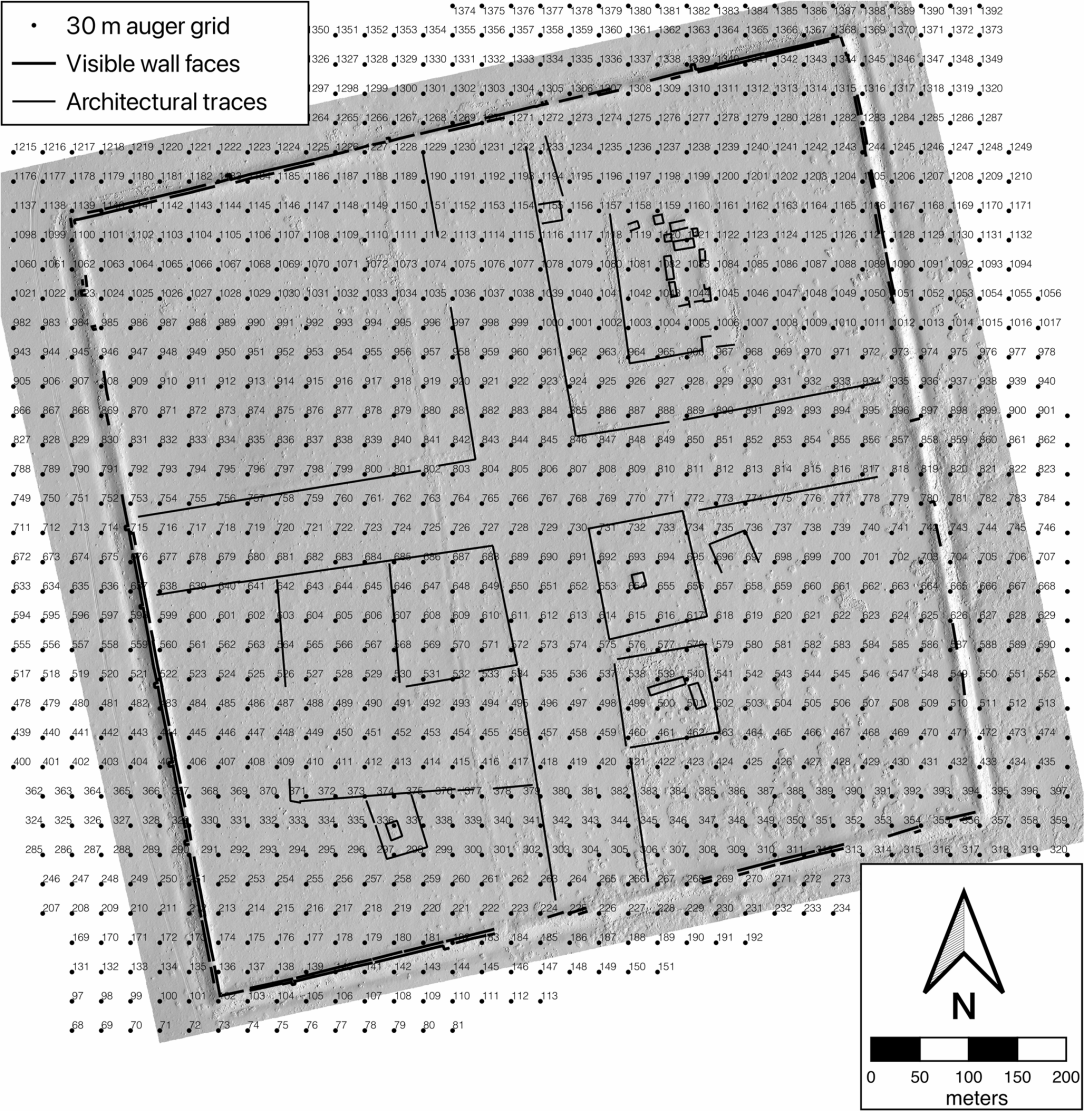
Shaded relief model of Baruun Kherem showing plotted auger collection points

**Fig. 3:**
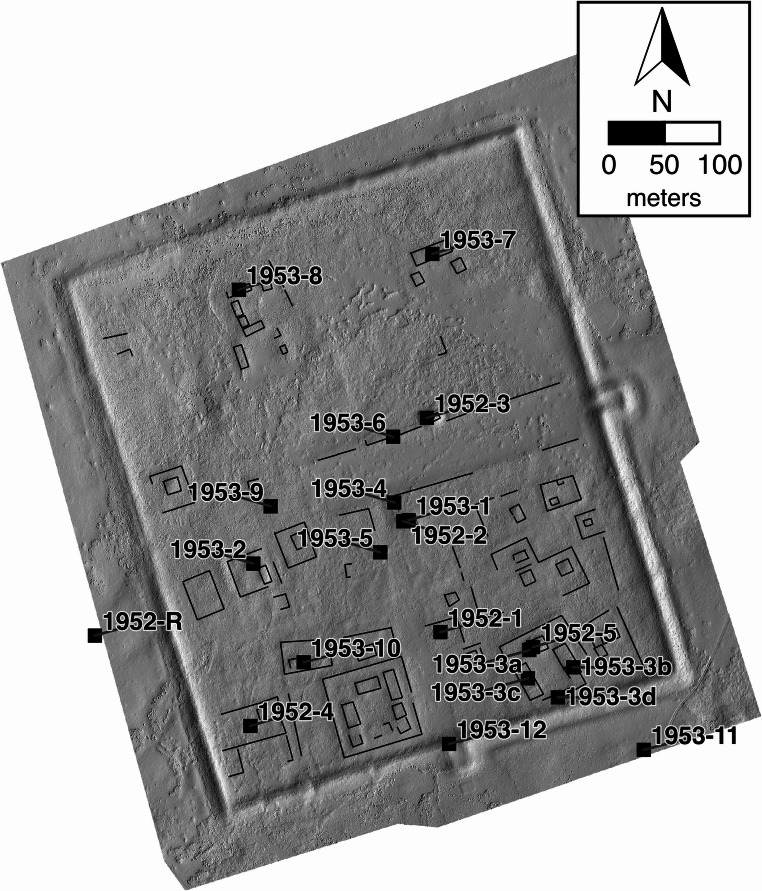
Shaded relief view of Zuun Kherem showing the locations of Perlee’s 1952 and 1953 excavations

This paper reports on the results of archaeological survey work at an urban site in Mongolia, Zuun and Baruun Kherem (ZBK). We offer a description of the archaeological finds and architecture, and address some key questions in the study of urban sites in eastern Eurasia. ZBK is made up of a pair of walled enclosures on the northern side of the Kherlen river in Murun Som, Khentii Aimag, Mongolia (47°13’57” N 110°22’14’ E) (Fig. 1). Named for their relation to each other, Baruun Kherem (BK) the “West Fortress”, and Zuun Kherem (ZK) the “East Fortress”. They are situated on higher ground overlooking inactive channels of the river around 1800 m from each other. Even a brief examination of the sites shows two key differences between the two sites. Firstly, their size - BK at 820 m square (67.2 hectares) (Fig. 2) and ZK is 510 × 430 m (22 hectares) (Fig. 3). Secondly, the BK enclosure is mostly empty of standing features while ZK features many mounds and a dense scatter of medieval period ceramics on the surface. This project investigated the interiors of the two enclosures and the area between them, we produced a photogrammetric map, carried out 1400 auger cores, located a ceramic kiln, recorded an extensive collection of ceramics and an unexpectedly large collection of fauna, as well as collected samples for a chronological study.

It is not any novelty as an urban site itself that makes the ZBK sites a valuable subject of archaeological examination. After all, towns, enclosures, central places, and vast cities have been built and inhabited continuously in the Mongolian steppe from the 1 st MBCE. onward, and an extensive body of literature has developed not only reporting on archaeologically investigation of such sites but also theorising on them (Perlee 1957c, 1961; Scott 1975; Rogers et al. 2005; Danilov 2011; Wang 2017; Kradin 2018; Bemmann and Reichert 2021; Reichert et al. 2022; Ishtseren et al. 2024). What ZBK offers is a chance to examine both inception and long-term dwelling in the towns and to define their dissolution, along with an obviously rich and relatively undisrupted archaeological record that can provide details of inhabitation. Finally, also, the possibility to compare two proximate and different enclosures.

The Kitan century in Mongolia was initiated with and characterized by the garrisoning of the steppe with fortifications such as ZBK. Our site-centered exploration of this period allows us to envision the Mongolian plateau not in terms of the extent of Liao territory and sovereignty, but as a vast pluralistic and contested frontier zone throughout its inhabitation and offering fresh insights and details into this poorly understood period.

### Project objectives and research aims

The archaeological approach to the ZBK enclosure sites aimed to establish the chronology and nature of their use from the artefacts and record their structural layouts. The enclosures had been previously examined and contextualized in the historical frameworks and they also are elements of debates about the nature of urban environments in medieval Mongolia. Here we will only touch upon the second of these, focusing instead on key archaeological questions about the enclosures themselves.

In addition to recording the extent and details of the two enclosure sites, the research carried out at ZBK aimed to examine several specific issues in the archaeology of medieval eastern Eurasia. A common characteristic of medieval walled enclosures and settlements in the region is that some of the enclosures appear to have no trace of architecture inside them and are considered by scholars to be empty enclosures. These empty spaces have been interpreted as places for temporary dwellings to be set up (Kradin 2018; Bemmann and Reichert 2021), where structures existed but left no traces above the ground (Duan 1963; Miller et al. 2019, Kradin et al. 2015), or as protected pastures (Shiraishi 2008). The ZBK enclosures are an ideal place to examine in detail for the first time the question of what happened in empty enclosures because BK is seemingly empty and ZK contains many building platforms and other features.

The establishment of chronology through artifacts and radiocarbon dating was one of our aims. We have already noted the parameters of Kitan Liao governmental involvement in town building during the 11th century CE. Historical sources and archaeological research (Kradin et al. 2015) have noted that Kitan towns could be built on the foundations of earlier Uyghur ones. Additionally, ZBK is in the same area of the later political center of Avraga which was active in the 12th and early 13th century CE (Kato and Shiraishi 2005). Finally, with the knowledge that non-urban medieval landscapes are accessible through archaeological survey (Wright et al. 2023, Reichert et al. 2022; Aherns 2016) we set out to record comparable surface archaeological and ceramic data to those surveys.

In addition to the basic question of activity within the enclosure walls, these sites can be examined in larger contexts. Kitan-Liao period centers in Mongolia are often thought of as purpose built administrative places focused on providing services for and controlling the surrounding regions (Kradin 2018). We examined the nature of administrative structures at ZBK using simple proxies for a power structure such as tile roofed buildings, elite material culture such as glazed ceramics and tiles (e.g. Perlee 1961; Reichart et al. 2022), and the spatial relationships of structures (Bemman and Reichart 2021).

With these aims in mind, we designed an intensive surface and subsurface sampling program in and around the enclosures of ZBK. We made use primarily of subsurface auger coring, a method not widely used systematically in Mongolia (though see Gardner and Burentogtokh 2018). Our methodology used a systematic and complete examination of the surface and subsurface of the sites and their immediate surroundings to search for buried middens or structural remains, covered ditches and dividing walls, or distinctive sub-surface cultural layers that could indicate inhabitation or animal penning in the empty areas. We also examined ceramics in sub-surface layers and on the surface of the sites to both study the number and density of material as well as contrast surface and sub-surface artefacts.

### Previous excavations

In 1952 and 1953 Khuduugiin Perlee, of the Mongolian Academy of Sciences, excavated 21 units in 12 areas inside the walls of ZK, as well as two outside the walls and one at the southern gate. These excavations concentrated on testing three of the large compounds, one of which was more extensively excavated in 1953. Examining the crossroads area at the center of the site and low platforms within several smaller compounds in the southwest of the ZK enclosure. In total c. 284 m^2^ were excavated (Fig. 3) (Perlee 1957a, b, 1961).

For the most part these excavations revealed horizontal layers including compacted clay soils, ashy soil fill, and wall debris. In some areas wall footings were located. Tile roofed structures were identified, as well as flat stone paved floors, stone and brick built *kang* heating systems, storage cellars, quern stones and mortars, pillar bases for large and small roof and wall supporting pillars. Many spaces contained ovens or hearths. Finds included faunal remains with cattle. sheep and goats dominating the assemblage. Greyware ceramic sherds in large amounts as well as white and green glazed wares were recorded along with iron objects including nails, wheel bushings, and plow blades. In all 5170 sherds of earthenware and 44 of glazed wares were reported, along with 2615 faunal bones.

Perlee’s interpretations of the enclosures turned to historical sources to seek to identify the site with a named settlement in the 1344 CE. History of Liao period, the Liaoshi. Subsequently other scholars, primarily historians, have made other suggestions about ZBK’s historical identity. Only Shiraishi has engaged with the archaeological remains as a factor in his interpretation of the site identity (Shiraishi 2008).

For the current project, these 20th century excavations provide a valuable comparison to test overall interpretations of stratigraphy and spaces. In a few cases auger cores are within a few meters of excavated areas, in most they are around 10–12 m. distant. Bottom depths varied between the methods in most cases. Auger cores sometimes were blocked by stones or soft sediment that excavations could go below, but also sometimes digging deeper than large area excavations. Overall, as will be developed below, the findings of these two projects are similar. Though there are some exceptions. Particularly, vastly more roof tile, brick fragments, and iron objects came from the excavations than from augers or surface collections.

## Data collection

**Fig. 4:**
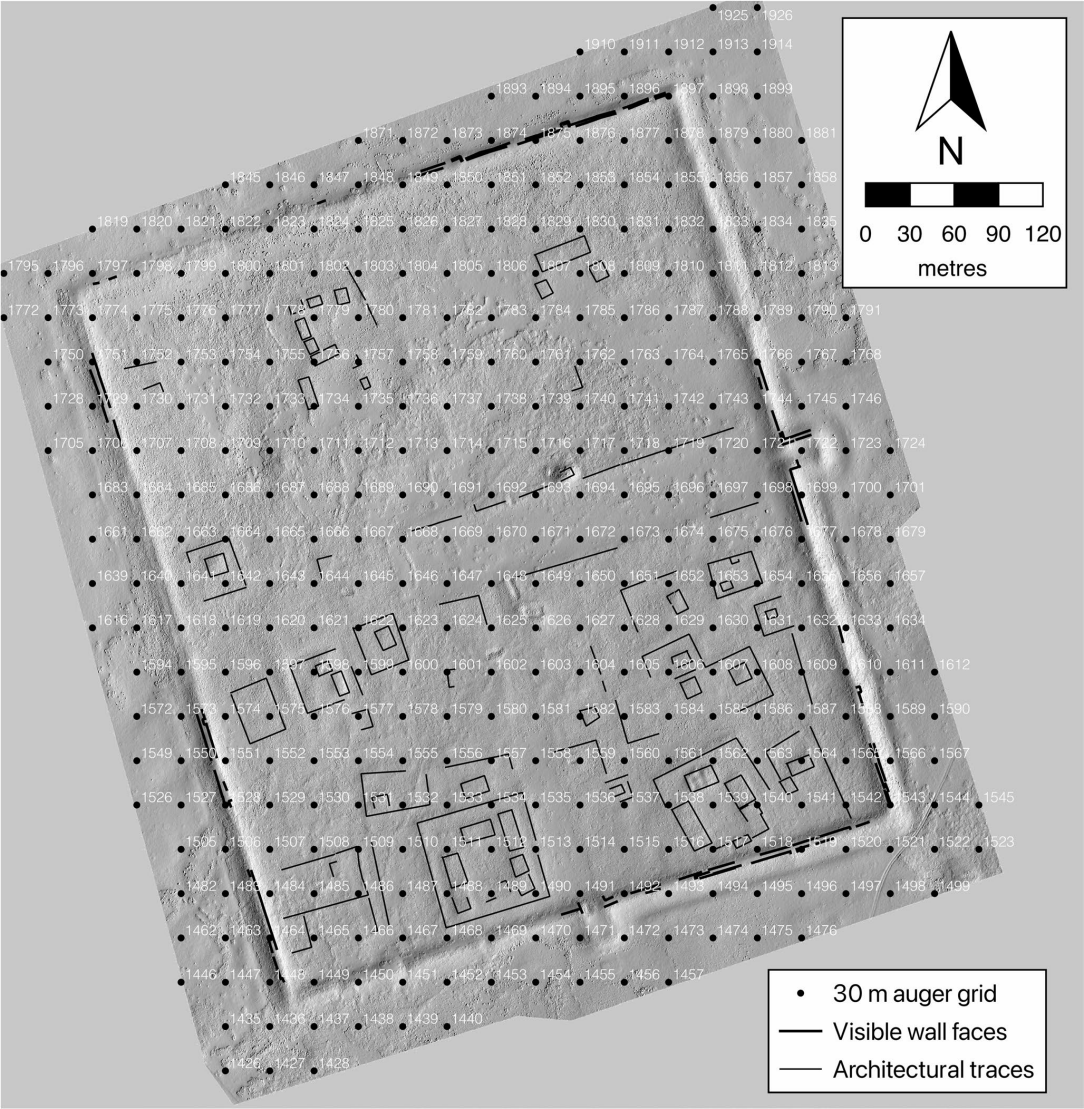
Shaded relief model of Zuun Kherem showing plotted auger collection points

The primary methods of the ZBK project were systematic subsurface artifact collection using hand augers (Howell 1993; McManamon 1984:257–259; Fry 1972). In addition we carried out surface collections in defined areas to compare with subsurface material and provide comparable data to other surface surveys. The basemap used for the collections was a high precision photogrammetric digital elevation model created from imagery collected by drone photography (Figure S1,S2,S3,S4). To prepare for the augering program detailed basemaps of a total of 177 hectares covering both enclosures of ZBK and c. 120 m. outside of the walls were created and a systematic 30 m grid of points laid out over the maps (Figs. 3 and 4). The grid pattern was created without reference to features or artifacts visible on the ground. Spacing of the grid was determined to balance worktime available and the visible scale of features in the sites. At this scale, one or more auger cores were placed within visible features but rarely were smaller platform mounds examined. The goal of this strategy was not to find details about structures, excavation is better suited to do that, but to find general trends across areas of the sites. Augering teams were guided to these collection points using hand-held GPS units with an accuracy of ± 2 m. The photogrammetric data was used to produce both a digital elevation model (DEM) of the enclosures and an orthomosaic image used to examine details of the structures and the contexts of the auger cores.

**Fig. 5:**
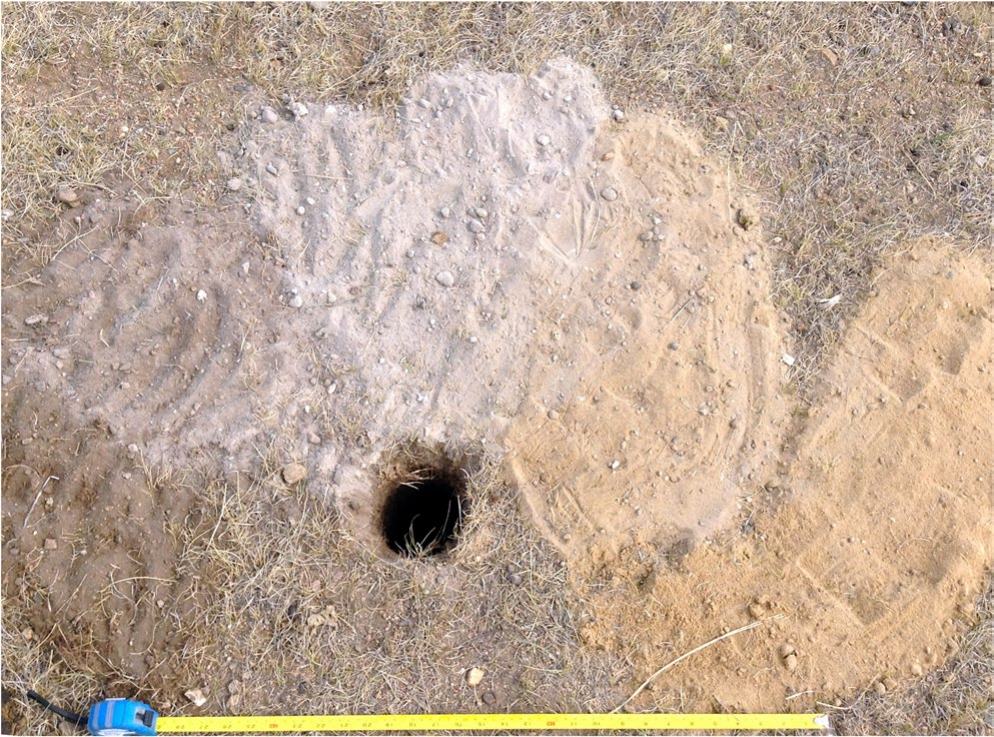
An example of the sediment spread from an auger core taken from point 1743 near the east gate of ZK. Moving left to right we see surface soil, into fine grey sediment, and finally loess. The depth at which a soil change was detected was measured with a hand tape. Each auger bucket was searched for artifacts

Augering work was accomplished in 10 days with 5–8 auger teams working simultaneously. Each auger team of 2–4 people carried a GPS with the collection grid loaded into it, a 10 cm diameter Quick-connect bucket auger with an additional 90 cm extension rod for deep deposits, a hand tape for measuring down-hole depths, and a tablet with a database interface for recording auger cores. Auger teams observed soil as it was brought up in the buckets and noted changes in color and texture as layers in their field records. Soil layer arrays were imaged using the tablet’s camera (e.g. Figure 5). Any artifacts from auger samples were tagged with auger point and (where appropriate) layer within the core. At the end of each workday, tablet databases were downloaded into a central database. In total 1400 auger points were tested over c.170 hectares and 3340 layers recorded. Of those points, 785 were within BK and 262 within ZK. The remaining 353 outside the walls.

Systematic surface collections were used within and outside the enclosures. These were 60 × 60 m squares, either built within the auger grid or measured when outside of it. 10 m^2^ ‘dogleash’ collections were used for extra detail within some squares. In total 49 60 × 60 m. squares were collected.

Documentation of ceramic finds was carried out in the field. All sherds were categorized by their fabric, thickness and form. Diagnostic elements were drawn and all assemblages were imaged. These methods follow those used by our team in several other regional survey projects (Wright, Honeychurch and Amartuvshin 2023, Wright 2010). Fauna was packed for analysis in Ulaanbaatar after the field season and a small sub-sample exported for radiocarbon dating. Charcoal was also exported for dating and species identification. All finds remain in the facilities of the Department of Anthropology and Archeology and the Archeological Research Center of the National University of Mongolia, Ulaanbaatar.

## Results

The results achieved by the auger survey of ZBK were beyond expectations. Work was completed quickly and effectively and a greater than expected amount of archaeological material was found.

### Soil profiles

Extensive augering produced a generalized profile of the soil columns in each site. The typical situation was a loose dark brown to brown silty sediment including small (c. 1 mm) gravels and overlying (at an average depth of 38 cm in BK and 48 cm in ZK) a light yellow brown to very pale brown reworked loess of fine silt with some clay and very few inclusions. Beneath this typical set of upper layers were deeper layers of clean sand, or mottled clay and gleys. This was not universal across the sites. Variation included the depth of each layer, the appearance of different colored layers (white, red or red-brown) and increased clayinesses of loess. These sediments were likely deposited during the Late Pleistocene when the Kherlen river was much larger and flanked by more oxbows and ponds than in historic times (Kim et al. 2017).

ZK’s layers are more heterogenous with loose brown soil and cultural layers atop loess and gravelly sand. This last being the composition of the elevated river terrace on which ZK was built. The section drawings of the 20th century excavations also show this variable soil profile in ZK, as well as nuances in the profiles such as compacted surfaces that augering did not recover. The excavations produced cultural layers as much as 170 cm below the surface in the central areas of ZK.

We observed several geomorphological and taphonomic processes in action at the site that have both hindered and helped our data recovery. Where surfaces are not protected, such as the tops of walls or much of the interior of BK, deflation has exposed sub-surface features and concentrated artifacts. In other areas windblown sediment and the vegetation that have taken root in it predominated. These dense siliceous grasses (*Stipa* spp.) obscure some areas of the site, but also have sprouted along the softer soils accumulating against mounds or in the joins between earthen construction elements. This provides a view of otherwise hidden structural elements. It is likely that water flowed across the interior of ZK from a natural breach in the northern wall towards the southwestern corner. This eroded a shallow channel and cut into walls and created pools at various spots. Finally, in more recent times animals have been housed near the sites and walked across them creating clear pathways and extra erosion of the walls as they crossed them.

The most visible transformation of the sites is the decay of earthen structures such as the enclosure walls. These once upstanding walls have slumped into mounds. Augering these mounds showed us that they first collapsed in large chunks creating mounds at their bases of material from the upper parts of the walls, they also were eroded, and covered with windblown sediments to become what they are today. These same processes were at play with platform mounds and smaller walls within the enclosures, which have completely decayed into barely perceptible linear mounds. Windblown and waterborne sediment has filled ditches, both inside and outside of the enclosures, sometimes to a depth of over a meter.

### Walls

Both enclosures are defined by their clearly visible walls. BK is square, while ZK is rectangular with its long axis N-S. The total length of the walls are c.3240 m for BK and 1875 m for ZK. Compared to the other Liao period walled sites, ZK is of a typical size, while BK is a large enclosure (Zhao 2019, Wang 2017).

In both enclosures the east gates are the most elaborately constructed. The east gate of BK had no extra upstanding earthworks around it, the entry was a straight path. However, that path was flanked by extra 10 m wide ditches. The east gate of ZK is behind a substantial berm that opens to the south and requires a sharp turn to enter the enclosure. Other gates or passages of the wall are visible. There is a clear southern gate at ZK with a barrier wall outside the gate can be seen as well as traces of a widening of the walls. In other areas visible depressions on the wall tops may indicate other smaller gates. Most notably on the northern side of BK, where the large avenue reaches the walls.

Using DEM measurements and images across the sites, the standing wall mounds at ZK are 2–2.5 m higher than the surrounding ground and the visible traces of original wall thickness are around 4 m. At BK the standing remains are 1.3–1.7 m and the walls c. 3.5 m thick. From excavations around the southern gate of ZK, Perlee (1957b) determined the walls there to be 4–4.2 m high, 3.5–4 m thick and made up of rammed earth layers 20–30 cm thick.

Many auger cores (65 in ZK, 82 in BK) were places on walls and in their ditches. Auger 1062 provides a particularly clear illustration of wall construction at BK. Examining the DEM of the wall at the same point, the likely base of the wall is 1.7 m below the current wall surface. The core was immediately at the visible exterior face of the wall and passed through a unit of red clay that was covered by typical loose brown surface soil at a depth of 1.7 m. This red clay was some of the first material to fall from the upper levels of the wall, landing against the base of the still standing wall and later being covered by other collapse and windblown sediment. The frequent occurrence of the same red clay across the subsurface and on visible wall tops of BK demonstrates that the soil used to build the walls was likely from the immediate area, probably dug from the ditches themselves and not specially prepared elsewhere.

Cross-sections can be drawn through the walls and the volume of fallen material piled against the walls can be measured and this added to the existing wall height to suggest the original height. Even considering that some of the accumulated sediment is deposited or removed by environmental processes, the rammed earth walls of both enclosures likely stood c. 2–2.5 m higher than they do today. This provides total heights of c. 4–5 m for ZK and 3.3–4.2 m for BK. With these measurements, In total, the walls of BK alone contain 42,500 m^3^ of rammed earth or mud brick, and those of ZK 32,750 m^3^.

The ditches visible outside of the wall today are c.1 m deep. Auger coring in the ditch bottoms, where there is a clear transition to loess soil visible in the core shows the possible original depth of the ditch bottom. At ZK (4 cores) this is c. 90 cm deeper than the modern surface. At BK (9 cores), the depth is c. 106 cm below modern surface. Making the depth of both ditches roughly 2 m below the local ground surface.

#### Wall chronology

At ZK there are several radiocarbon dates that focus on wall building. These dates were incorporated in the larger phase model of dates from ZBK using Oxcal 4.4.4 (Bronk Ramsey 2001, 2009, 2021) Core 1618 contained a deeply buried bone (210 cm below surface) c.1.5 horizontal meters from the interior of the wall on the western side. This bone’s calibrated modeled date is 1006–1033 CE (SUERC-111179, Fig. 9,table S3). The bone was found in the upper part of a mottled clay layer that can also be found outside the wall (in auger cores 1617, 1593) at a similar elevation. At point 1618 this layer was covered by 110 cm of fine gray midden and loose soil, later accumulation against the wall face. The low stratigraphic position of this bone, on a likely non-culturally modified surface, suggests that it is dating the earliest period of the wall. Next, a large charcoal sample from core 1744 is in a comparable position, immediately at the wall interior and below the fine grey midden soil, on the eastern side of ZK, this also dates to 1006–1033 cal CE (UBA-50370, Fig. 9,table S3). These dates suggest the early 11th century CE for the period when the ZK wall bases were exposed, and presumably the walls newly built. A date near the bottom of the ditch (core 1789) suggests that the ditches could have been open and maintained for up to 140 years after that.

### Architecture and street plans

Both enclosures are oriented 7–8° west of the geodetic north. In addition to their walls, the enclosures are dominated by wide avenues and platform mounds that could have supported buildings or temporary structures. The mounds appear to be made from compacted reworked loess soils atop the original ground surface.

What is immediately obvious in aerial images of both enclosures are wide avenues oriented perpendicular to the walls. In both sites these have prepared surfaces and features defining their edges. The most obvious example is the cruciform avenue of BK. This is c. 100 m wide and crosses almost at the exact center of the walled enclosure. It is defined by c. 10 cm high 6–10 m wide features that could be the remains of a wall or low mound. In many auger cores across the avenues, a layer of angular gravel or sand was noted suggesting a prepared road surface. The avenues are oriented rotated c. 10° N and W which makes the eastern arm perpendicular to the wall at the east gate of BK. This cruciform avenue structure is seen at other Kitan-Liao sites in Mongolia and Inner Mongolia (Wang 2017).

A similar avenue can be seen at ZK. It goes from the east gate to just beyond the approximate center of the site. It is also oriented perpendicular to the wall at the east gate. This avenue is c. 50 m wide and flanked by raised mounds 8 m wide and 40 cm high for much of its length. Its surface seems to have been cleared down to the gravelly sediment that underlies much of the site. A smaller avenue, 22–25 m across and flanked by structural compounds runs southward from the center of ZK to the south gate. Finally, there is a possible trace of a northern avenue offset from the central crossing. This is seen as a 21–23 m. wide linear depression where two of nine auger cores noted pebbled sand and clayey soil layers near the surface. ZK also contains outlines of possible smaller streets defined by compound boundary walls, where these can be measured they are 8–10 m across.

Using the DEM and orthophotos collected during the initial mapping of the site we were able to draw outlines of various structures at ZBK (figure S3,S4). This mapping was an interpretational process accomplished by looking at areas of higher ground, changes in soil color, and vegetational linearity as *stipa* spp. grasses have grown into areas of softer sediment and wind blown sand against structural traces. Roof tiles found by the survey helped to define some structural areas. Imagery was, in some places, able to show traces of mud plastered wall faces or construction blocks that provided guidance for less precise mound features and soil colors.

The many traces of platform mounds and walls in ZK give a sense of the architecture of the site. Platform mound features may have been actual platforms for perishable structures or the remains of earth walled structures that have collapsed into mounds. Our data does not allow us to gauge the original height of any structures. Though with platform mounds, precise digital elevation model variation shows that they stand between 30 and 60 cm above the surrounding ground in ZK. The larger structures in BK stand 0.8–1.3 m high at present.

There are three basic types of structures visible: linear features, compounds, and large compounds. Linear features or mounds could be partial compound walls or stand-alone walls. Compounds are the most numerous structures and are typically an associated group of one or two platform mounds and an enclosing wall. In the best preserved cases, the enclosing walls’ longest dimensions are c. 30 m, the compounds are c.800 m^2^, and the platform mounds cover 50–150 m^2^. Traces of further construction (tiles, pillar bases, or bricks) were rarely found in these enclosures. The main exception being the compound area between points 1664/1642. Which is also on high ground within ZK.

Large compounds include traces of construction and contain many platform mounds (2–8 in the ZBK examples). There are two of these in BK and three in ZK where enclosure walls can be clearly traced. There are two more possible large compounds in the north of ZK defined primarily by groups of platform mounds. In the more crowded enclosure of ZK, large compounds are between 60 and 70 m on their longest side (c. 4300 m^2^). In BK, the large compounds are 105 by 170 m and 90 by 90 m and have been built without the need to conform to or provide for surrounding structures. The layout of the large compounds show several clear details. All form enclosing courtyards, and in several cases small gatehouses on the southern sides can be seen. The largest platforms stand at the northern edges of these courtyards. Gaps in wall mounds may show secondary entrances.

Only one of these large compounds in ZK has been excavated, enabling us to compare augering interpretations to excavation. In 1952 Perlee dug a trench (Figs. 3 point1952-5) bisecting the northernmost platform mound, the principal structure of the compound. In 1953 four other excavations in the principal structure were carried out in the east and west ranges, and the entrance to the structural group on the southern end (1952-3a-d). Vast numbers of roof-tile fragments were found along with stone pillar bases buried beneath wall collapse. The area of the principal structure were c. 92 m^2^, (Perlee 1957a, b) c. 40% the area of the visible platform mound today. Tests of other structures showed they were of similar length, but narrower width, to visible platform mounds. Only two auger cores were made on the platform mounds of this compound (1540 and 1517) these showed no remains of construction debris, however, loose brown sediment, sand, and fine grey soil containing a few artifacts was found from the ground surface to a depth of 90–120 cm. Topographic variation suggests that the platform mounds are up to 1.1 m higher than the original ground surface, but with much accumulated sediment around them. This case again shows the difference between surface and auger study and excavation, though both reach similar conclusions about the platform mounds, excavation provides more accurate information about the structures.

A datable sample was recovered from the enclosing ditch of the larger of the two BK compounds (point 1122, table S3). The ditch was infilled with homogenous loose sandy soil to a depth of 200 cm. This appears to be infilling of the ditch by wind and waterborne sediment. Bone fragments and ceramics were recovered from the auger core at 125 cm and 180 cm below ground surface. The bone at 180 cm was a fragment of worked bone. A dated bone sample was taken at a depth of 125 cm. This date is calibrated between 970 and 1040 cal CE. Showing a time when this ditch was an active feature, but had been open for enough time for an accumulation of sediment in its bottom.

Not every area within the enclosures contain visible platform mounds. This includes most of BK and the northern half of ZK. Though some of these areas contain windblown sand, thick stands of grass, or deflated surfaces, augering demonstrated that there are no signs of buried structures or cultural layers in these areas.

#### Tile roofed structures

Ceramic roof tiles were found in both enclosures. Tile roofs are commonly interpreted as the remains of ornate buildings associated with administration, elite residences, or religious structures. However, all tiles found were plain, with similar fabric impressions from their manufacturing process remaining on the interior of the tiles. No end-caps and few decorated tiles were recorded, and those all near the large compound in the northwest of ZK. The tiles were densely concentrated in defined areas across both sites, found on the or around the large compounds and usually as dense mats of broken tiles suggesting that they fell in place from roof to ground surface.

We can speculate on the nature of structures based on the tiles found in both enclosures. In BK, tiles were found on all the platform mounds. The mounds at points 500/540 also had a visible stone pillar base, suggesting a taller roof and more imposing structure. The smaller, more central mound north of that taller mound contained many tiles and may have been a religious building because of its shape, central location within the site, and the symmetry of its compound wall with a wall to the east of it. In ZK five areas of dense surface tiles were recorded. Four of these sites are large compounds. The fifth area is a smaller compound with single tiled structure on high ground at the western edge of the site on the central E-W axis. By its location and tiled roof, it is possible this building could also be a religious structure.

The presence of tiles without mounds can be used to make a case for additional tile roofed buildings outside of large compounds. Perlee’s 1952 excavations at point 1485 (1952-4), in the southwest corner of ZK, contained a quantity of roof tile fragments and a fragment of a pillar base all in the top 50 cm of the ground. Perlee interpreted this area as the remains of a tile roofed building. The cores in this area (1485, 1486, 1465,1466) all contained artifacts and several stopped on stoney layers at 55–70 cm depth. Linear features are visible in orthoimagery defining enclosures up to 60 m on a side. Together these data suggest that this corner of the enclosure may have been a fifth large compound. Excavations show subsurface tile fragments in some places though augering did not recover any.

### Cultural layers

Augering revealed a pronounced cultural layer at ZK. A widespread layer of sediment consisted of a light brownish gray very fine silt with slight clayey plasticity and 20–30% inclusions of small round gravel of less than 1 mm with some larger sub-rounded gravels. This layer was found in 45% of the cores within the walls. The co-occurence of archaeological materials and fine gray soil in the auger cores is extensive and suggests that this soil is the product of inhabitation. 70–80% of the artifacts found in ZK came from fine gray layers.

The fine gray soil layer rich in faunal remains, pottery and small burnt fragments that is found across most of the built up areas of ZK can be interpreted as the residues of waste disposal by residents in the town and the post-depositional reprocessing of that material (a transposed primary deposit [Schiffer 1987]). Marks of carnivore chewing, likely by dogs, were found on five faunal specimens from ZK (Table S2). Taxa and skeletal elements varied, and all but one were found in the areas of fine gray sediment.

Perlee’s excavations also describe an ashy soil with animal bones and sherds across ZK. In each case, where an excavation found this layer (Figs. 2 and 1952-1,1953-1, 1953-4, 1953-9), the nearby auger cores also found it. Only in excavation 1953-9 were the depths similar. In the other excavations nearby auger cores reported fine grey soil at higher depths than it first appeared in the excavations. In these three cases we should consider that the excavations were all begun on low mounds, where the auger grid placed many cores off those mounds. The c.20 cm elevation differential between the starting points brings the layers to the same general depth.

These easily interpreted cultural layers of ZK help to interpret inhabitation layers at BK. There are almost no traces of small platform mounds there, however three auger cores in part of the northeastern quadrant of the enclosure revealed fine gray layers, some with ceramics and bones. However, the strong association between fine grey soil and other archaeological material seen in ZK is not the same in BK, with only one area, near the center of the north wall (around points 1271–1272), having both fine gray soil and sherds, and the layers are not as thick (average 24.6 cm at BK compared to 37.7 cm at ZK). All of this suggests a less intensive occupation of the BK enclosure.

This artifact rich layer corresponds generally to areas with visible structural remains. These deposits are thickest in areas that can be considered exterior spaces — outside of compound walls, between the main walls and structural traces, alongside street intersections, and are thinnest or not found inside large compounds, in areas with no structural traces, or in avenue areas. The fineness of the sediment and small inclusions suggests repeated compaction of soil in areas sheltered from wind. Conditions that could be expected in the streets and courtyards of a densely built up town. Though a more detailed geoarchaeological study is needed to fully demonstrate this.

Taken together, these factors allow these layers to be interpreted as depositions during the time of inhabitation of the town. Larger compounds, likely with restricted access, and large streets were kept clean, while smaller and less regularly used spaces both received waste, and were sheltered, but also sometimes trampled, producing fine sediments with bone and pottery fragments.

### Ceramics

The ceramic forms recovered are overwhelmingly the typical Kitan-Liao period grey wares — fast wheel thrown jars and bowls of different types (Kradin 2018; Kradin and Ivliev 2008, Makino 2007). Our focus here is primarily on diagnostic and glazed sherds (a total of 182 sherds). There are two working assumptions about how ceramics were deposited into the archaeological record at ZBK. First, there are some areas of dense sherd deposition outside the walls that could be structured disposal areas. Second, waste is deposited within the walls and most frequently outside of compound areas. We assume long term ad hoc disposal proximate to use areas.

A previous study of form and decoration of sherds from the 20th century excavations (Battsetseg 2009) was carried out on a selection of the 280 earthenware sherds stored at the Institute of Archaeology in Ulaanbaatar. The major forms identified included deep bowls, short neck and long necked jars, dishes, and basins with flat and convex bases. Types similar to the 21 st century collections. Of this non-random sample of sherds studied, 70% were decorated with angular punctates and rolling stamps, and 37% of the rims showed possible elements for lids.

**Fig. 6:**
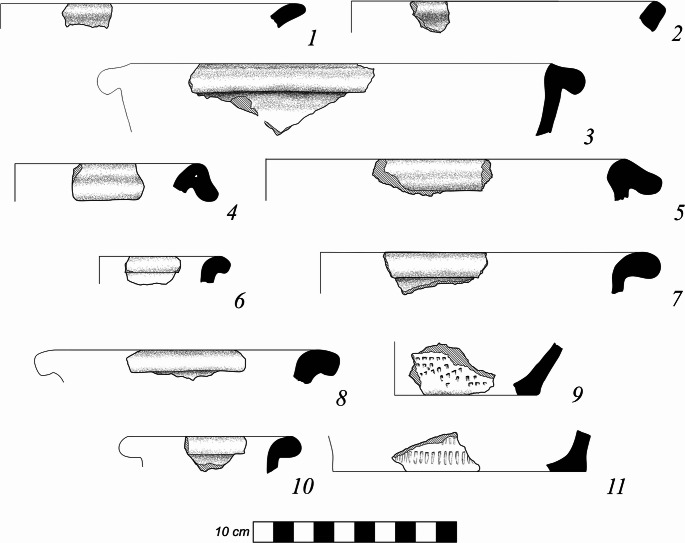
Diagnostic earthenware sherds collected at Zuun Baruun Kherem. 1–2, steamer or large open mouthed jars. 3–5, drop-rimmed jars. 6,8–9, everted rim closed mouth jars. 7, large jar. 10–11, tall bottle jars. Drawings from Misterkiewicz 2024

**Fig. 7:**
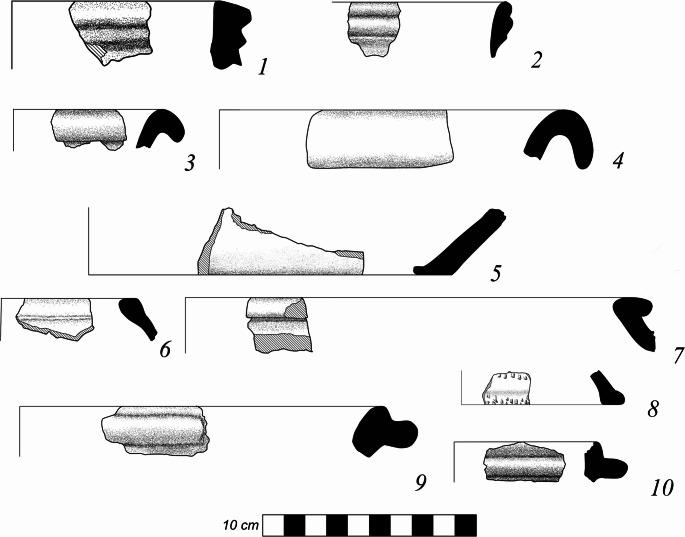
Diagnostic earthenware sherds collected at Zuun Baruun Kherem. 1–2, corrugated rim pots. 3–5, basins. 6–7, inverted rim jars. 8, lid. 9–10, lidded vessel rims. Drawings from Misterkiewicz 2024

During the ZBK project a total of 188 bags of ceramics were collected from auger cores (Table S1) and 81 from surface collections. Every sherd recovered was examined and its basic data recorded. A total 2376 sherds, tile and brick shards, grinding stone fragments, and metal objects were recorded of which 133 were diagnostic rim and base sherds (Figs. 6 and 7). The vast majority of the sherds are grey to grey-brown earthenware and 43% of those are decorated, with the large majority of them having angular punctate decorations. Some complex and roller stamps, ‘cogwheel’ stamps and burnished pattern designs were found. The typical dominant ceramic forms of the medieval period are found throughout the ceramic assemblage. This includes various drop-rimmed jars and narrow-necked jars, close-mouthed jars, wide-mouthed deep pots with heavy corrugated rims, and larger jars with small rims (Fig. 6:3–9,7:1–2). Overall, this is a typical Kitan period ceramic assemblage for central Mongolia. Table 1. shows the counts of vessel sherds from the different collection types and areas. The large area surface collections yielded many more sherds than the smaller and more targeted auger cores.

Some forms are found evenly distributed around the ZBK sites. Primarily, these are open mouthed jar sherds with heavy corrugated rims, that in other contexts has shown signs of being used for cooking (Kradin and Ivliev 2014:156) and everted rim jars, a very common form of medieval vessel, which were found in all areas that contained ceramics.

**Table 1:** Sherd counts from auger and surface collects at Zuun Baruun Kherem

	BK	ZK	Exterior	Total Sherds
From auger cores	43	348	38	429
From surface	277	881	630	1788
Total sherds	320	1229	668	2217

In addition, within this ceramic assemblage there are two locationally distinct sets of vessel forms. Large jars, interpreted as storage vessels, were found near many of the compounds in ZK and BK. These have been found set into ground at other urban sites (Kradin et al. 2011).

Other large diameter non-jar rims that are possible steamer vessel rims (Kradin 2018:157) follow a similar pattern. Though no perforated steamer base sherds were found. Together these suggest household functions such as food storage and preparation. Basalt quern stone fragments, another important element of food preparation, were found on the surface of ZK (*n* = 8) in all areas of the site where concentrations of sherds were also found. 

An additional vessel type that is identified at many locations around ZK (12 sherds in 8 locations) are basins. These vessels have a diameter of 50 cm or more, a heavy down-turned rounded rim, and sharply angled bases with a diameter of around 40 cm. Vessels of this size are not easily transportable and may also have been household features. Basins are one of the clear differences between the ZK and BK enclosures. However, the difference between the two assemblages appears to be driven more by the difference in possible use of the spaces. In the areas of BK that have platform mounds and fine gray soil layers, the ceramic assemblage fits with that of ZK’s similar areas. In BK outside of those areas, the ceramic assemblage resembles those found outside the walls of ZK, dominated by smaller storage and cooking vessels.

Tall vessels, a distinctive type of Liao period storage jar recognised from bases, rims and body sherds, are found frequently outside of the walls and in BK only, a different use pattern than ZK’s household ceramics. This distribution is also similar to the ledge-rimmed cylindrical vessels interpreted as lidded vessels.

Glazed wares make up about 2% of the ceramics collected across the two sites (*n* = 49 pre-modern glazed sherds). These are primarily divided between the common dark brown or black glaze that is the most difficult to chronologically locate and equal parts green glazed stoneware (*n* = 10) and cream glazed stoneware (*n* = 13). These are all the common glazed wares of Kitan sites. Single examples of exceptional glazed wares including a piece of possible southwest asian green glazed earthenware were found outside the walls of ZK. More colorful glazed wares were also found outside the southwestern walls of BK perhaps indicative of a re-use of the area during later medieval or Mongol empire times when colorful glazed ceramics were more widespread than during the Liao era.

Comparing the ZBK glazed ware assemblage to a non-urban Kitan-Liao one we see general continuity. Data from the survey of Baga Gazaryn Chuluu, in a similar semi-arid environment (Wright et al. 2007, Makino 2007) shows 3.9% of the sherds from medieval sites there are glazed wares. However, there are proportionally more dark glazed sherds, which are the most uncertain chronologically. Of the other main glaze types, cream ware and green, there are approximately equal proportions of those glazes, just as there are at ZBK.

In sum, though most of our diagnostic sherds are based on surface collections rather than secure contexts from augers, it is possible to recognize a ‘town set’ of household vessels, lamps and grinding stones. And an ‘outside town’ set including primarily smaller cooking and storage jars, narrow tall storage jars, and a few colorful glazed wares. This spatial diversity suggests both possible chronological differences between the use of areas as well as distinct habitation styles in different areas.

#### Kilns

Though Perlee observed metal working debris during his recording of the site (Perlee 1957a, b), the current survey did not locate any metalworking remains. The primary evidence of industrial processes that were recorded are ceramic production. A kiln site was located by walk-over survey 430 m to the southwest of ZK’s walls. The ground surface showed dense black charcoal rich soil and ceramic sherds, a group of closely spaced auger cores were carried out here. Recovering kiln construction debris, charcoal and many sherds in the 100–135 cm of soil above loess and gravel. This suggests a kiln dug into the ground, built up, and surrounded by detritus from its firing and cleaning. Many sherds from this area were sharply broken and without worn surfaces or edges. A fragment of salix sp. charcoal dated between 960 and 1060 C.E (UBA-50375) was recovered here. It is likely that this was not the only kiln in the area. A similar dark soil location was found 60 m west of the first and several dense ceramic deposits were recorded in Perlee’s excavations outside the southern wall of ZK (1953-11) and in surface collections 600 m south of ZK. The high relative sherd density of these areas suggests they could be dumps for unsuccessful ceramics from the kilns.

### Fauna

Despite the limited scale of the contexts available through auger coring an unexpectedly rich faunal assemblage was found at ZBK. A total number of 337 zooarchaeological specimens were recovered. These are divided between the two enclosures, with many more in ZK (*n* = 312) than BK (*n* = 25). The identified taxa were those expected for domestic livestock husbandry and wild fauna in this environment.

Of the 25 identifiable specimens from BK, eight were identified to taxon covering the three most common domestic livestock taxa of the Eurasian steppe, *Equus* sp. (*n* = 2), *Bos* sp. (*n* = 1) and *Ovis/Capra* (*n* = 5) of which one was further identified to *Ovis* sp. In the *Ovis/Capra* group two phalanges provided epiphyseal fusion data, indicating the age at death of at least one individual younger than 12–18 months. Two smaller fragments were identified as Caprine/Antilopini as the presence of gazelles in the assemblages could not be ruled out. The rest were medium sized and large mammals (*n* = 9). The BK sample covered all parts of the skeleton.

There were unexpected findings regarding faunal processing in BK. Given the small sample size from BK, the proportion of specimens (4 of 25) exhibiting signs of processing is relatively high (Table S2). Two *Ovis/Capra* specimens, a rib fragment and an astragalus showed cut marks corresponding with slaughtering or meat processing. Two others (long bone fragments grouped as medium and large mammals) were cleanly sawed through, which might indicate either a more systematic dismembering process or preparation for bone working. Both of these were found at point 1122, the infill of a large compound’s ditch.

**Fig. 8:**
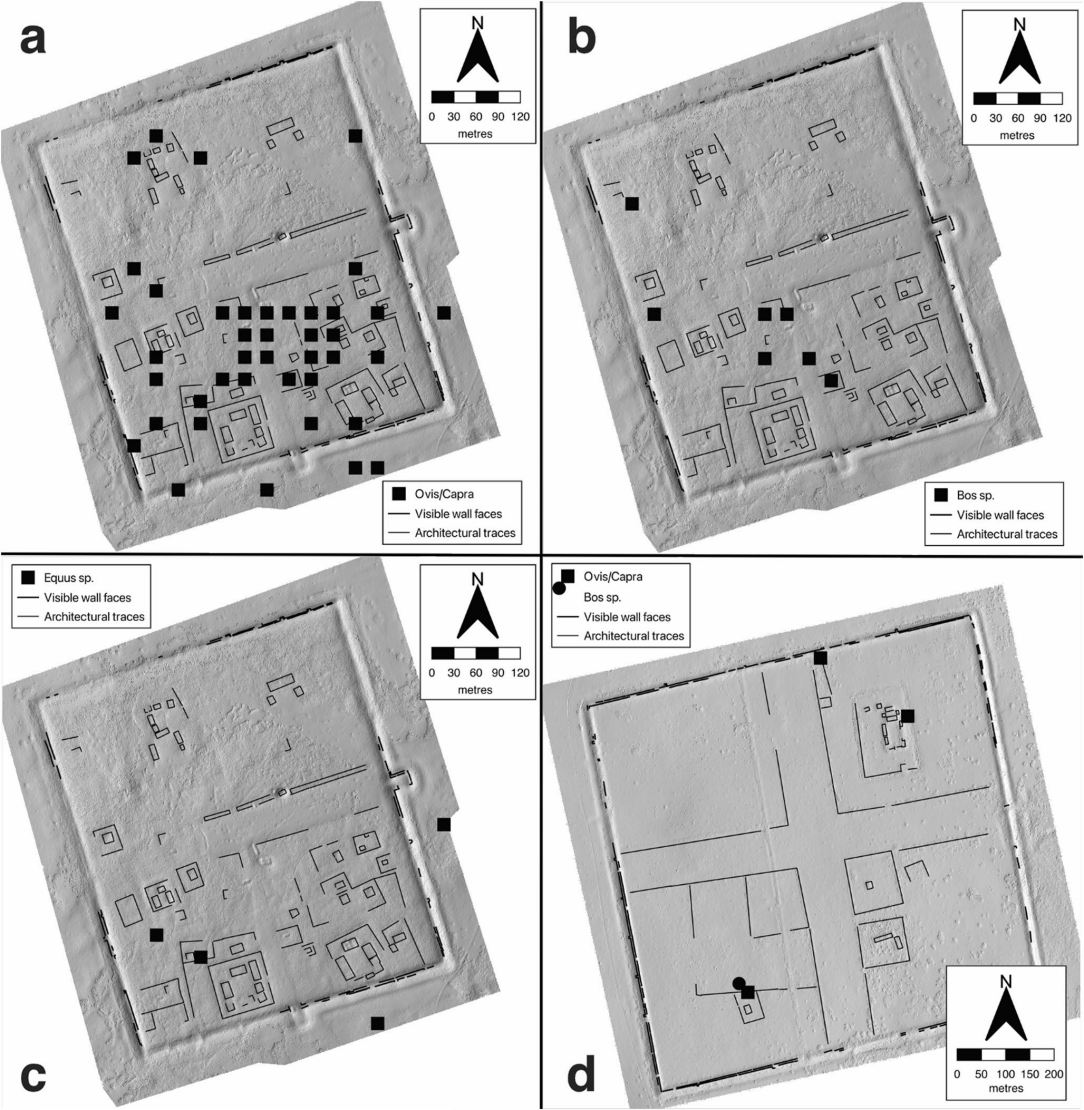
Faunal species distribution at ZBK.Points show presence or absence of the species in that collection point. A, *Ovis/Capra* in Zuun Kherem, B, *Bos* sp. In Zuun Kherem. C, *Equus* sp. in Zuun Kherem, and D, *Ovis/Capra* in Baruun Kherem

For the zooarchaeological remains recovered from ZK, it was possible to identify more specimens and compare parameters across the sampled area (Fig. 8). Of all specimens obtained at ZK, 29% (*n* = 90) were identified to taxon, 20% (*n* = 63) were grouped as small/medium/large mammals, and 51% (*n* = 159) were unidentified fragments. Most identified elements, and the largest fragments, are concentrated in the area defined by points 1624 − 1560, the central area of southeastern ZK, while many unidentified fragments are found in the periphery of that zone and the northwestern quadrant of the enclosure where structural mounds are visible. The distribution of skeletal elements appears to be heterogeneous. Skeletal portions rich in meat (limbs and vertebral column) versus skull and extremities were not found in different spatial areas but largely overlapped without clear distinction. Three specimens exhibited cut marks, an *Ovis* sp. metacarpus, an *Ovis/Capra* humerus and a *Bos* sp. centrotarsale, all are possible examples of separating extremities from more meat rich portions as part of food processing.

Of the identified zooarchaeological remains, *Ovis/Capra* was the most abundant group with 66% (*n* = 59). Of these seven were further identified as *Ovis* sp. and one as *Capra* sp. Nine specimens were recorded as Caprine/Antilopini, which given the lack of securely identified gazelle remains in the material also likely belong to the *Ovis/Capra* category. Demographic data for sheep and goats provides information on the economy of ZBK. The age at death is based on epiphyseal fusion and morphology. Four specimens indicated a kill-off at a younger age than 12–18 months, three within 6–12 months and one within 0–6 months based on epiphyseal fusion data. Together with two specimens, which based on morphological traits were identified as fetal remains, this provides evidence for local breeding of sheep and goats, and perhaps penning within the town. In a larger assemblage these data would suggest the utilization of sheep/goat herds for dairy. Second most abundant taxon was *Bos* sp. with seven specimens. Three specimens with epiphyseal fusion data indicated an age at death older than 7–10 and 18 months respectively. *Equus* sp. was represented with seven specimens at five collection points.

The ZK faunal assemblage yielded a few noteworthy singular finds. A phalange identified as *Camelus bactrianus* was completely preserved deep in core 1618. A carpometacarpus and a scapula of a small avian were found in sample 1470, and a fish bone, identified as the pectoral spine of a *Siluris* (likely *asotus*) was found outside the east gate in 1724. These three were all found in the periphery of the site and each very close to the city wall.

Focusing on the fauna of two spatially distinct distributions we see possible traces of difference in the deposition. The central southern area 1624/1560 is an area c. 5000 m^2^ that has both no well defined structures, thick fine gray soil, and the largest concentrations of sherds and bones. Though it is tempting to think of this as an important midden, 20th century excavations around this area followed cultural material to over 2 m below the present ground surface (Perlee 1957a, b). Combined with the natural topography beneath the built areas this suggests that this area of ZK was lower elevation and most platform mounds buried by sedimentation. Even today, this area is 0.8–1.0 m lower than adjacent areas.

Counterbalancing the infilling of part of the settlement with sediment, the 1674/1607 structure group in the southeast quadrant of ZK produced many ceramics both in subsurface and surface collections. Also a relatively dense selection of burnt bone fragments. The intensive surface collection that spanned this area contained 356 sherds, more than any area inside the ZK walls. Though the diagnostic assemblage here is not exceptional, consisting of the typical forms with dropped-rims the most common. This area shows the traces of 3 or 4 compound groups. And no sign of major post-abandonment surface modifications.

All in all, the faunal sample from Zuun Baruun Kherem provides a manifold set of information given it has been obtained from auger coring only. As might be expected for a central Mongolian medieval community the material indicated both local herding action as well as activities for quotidian household subsistence provisioning and processing. Some of the remains were clearly disposed of and scattered on the surface which evokes an image we know from rural townscapes in regions where pastoralism is practiced and where bone fragments are everyday objects.

### Wood resources

Wood resources are a complementary data set to faunal remains. Both illustrate the surrounding landscape through finds within the walls. Nine large charcoal samples were found in the auger cores of ZK. Three were alder (alnus sp.), three birch (betula sp.), one pine (pinus sp.) and two willow (salix sp.). One charcoal fragment from Perlee’s excavations (1953-8) was identified as birch. One of the willow samples dated to recent times. Dating of six samples showed no chronological divisions of wood types being used. The majority of this wood would not have been found immediately around the towns. The implication is that if this was firewood, people were bringing their larger logs from the hills 20–30 km to the north of ZBK, and rarely harvesting from the nearer riparian woodlands along the Kherleen river.

## Coins

Four coins were found in and around ZK, two within the walls and two outside the walls (Table 2). All are single sided copper alloy coins, three of which date to the eleventh century, produced in the Northern Song (960–1127 CE), and one to the tenth century Southern Tang regime (943–959 CE). This is not the first time coins have been recovered from ZK, Perlee reports finding coins though does not provide details on the specific coins (Perlee 1957b). In the wider archaeological context of Mongolia, such coins from the Song have been found in urban sites such as Chintolgoi Balgas (Kradin et al. 2011) and also by archaeological survey in small rural sites around the Delgerkhan uul (c.120 km southeast of ZBK) (Amartuvshin, forthcoming). The four periods of coins found at ZBK have also been excavated in tombs and collected on the surface of walled-town sites across the Liao empire (Zhongguo Qianbi 2006, 690–709). What can be said of these finds in their context is that similar coinage was circulating, albeit at lower levels, throughout Liao dominions in Mongolia both in urban and rural communities. Lastly, if coins are thought of as indicative of everyday economic activity, comparing ZK to BK it is ZK which clearly had a higher frequency of commercial activity than BK.

**Table 2:** Coins recovered by the ZBK project. All coins were found on the surface in and around Zuun Kherem

Coin	ZBK SF	Coin	Date Range (CE)	Diameter (mm)	Square hole width (mm)	Thickness (mm)	Weight (g)	Reference
1	11	Xiangfu Yuanbao 祥符元寶	1008–1016	25.2	5.2	1.2	3	Zhongguo qianbi 2005:54
2	15	Tangguo Tongbao 唐國通寶	943–959	24.5	5.6	1.2	3	Zhongguo qianbi 2003:639 − 45
3	17	Jingde Yuanbao 景德元寶	1004–1007	24.3	5.6	1.3	4	Zhongguo qianbi 2005:46–48
4	15	Jiayou Tongbao 嘉佑通寶	1056–1063	22.9	5.9	1	4	Zhongguo qianbi 2005:143–147

## Radiocarbon dating

From the collections at ZBK 20 organic samples (bone or charred plant remains) were selected for radiocarbon dating. In all, 15 samples from ZK, 1 from the exterior near ZK, and 4 from BK were dated (table S3). The materials submitted for analysis were chosen to examine the construction, use, and abandonment of the two enclosures, particularly any temporal separation that can be seen between the two. There were not enough samples from individual large enclosures to examine them specifically.

The resulting radiocarbon ages were calibrated and phase modeled in OxCal Online 4.4.4 (Bronk Ramsey 2001, 2009, 2021) using the IntCal20 calibration curve (Reimer et al. 2020). The area of the radiocarbon calibration curve where Kitan activity occurred is relatively flat (Reimer et al. 2020) making chronometric precision difficult. One date, SUERC-111,189, had an uncalibrated radiocarbon age of 256 ± 25, much later than the periods being focused on and was removed from further study. Some specific results of these dates have been discussed above, here we consider dates from each enclosure as a whole.

**Fig. 9:**
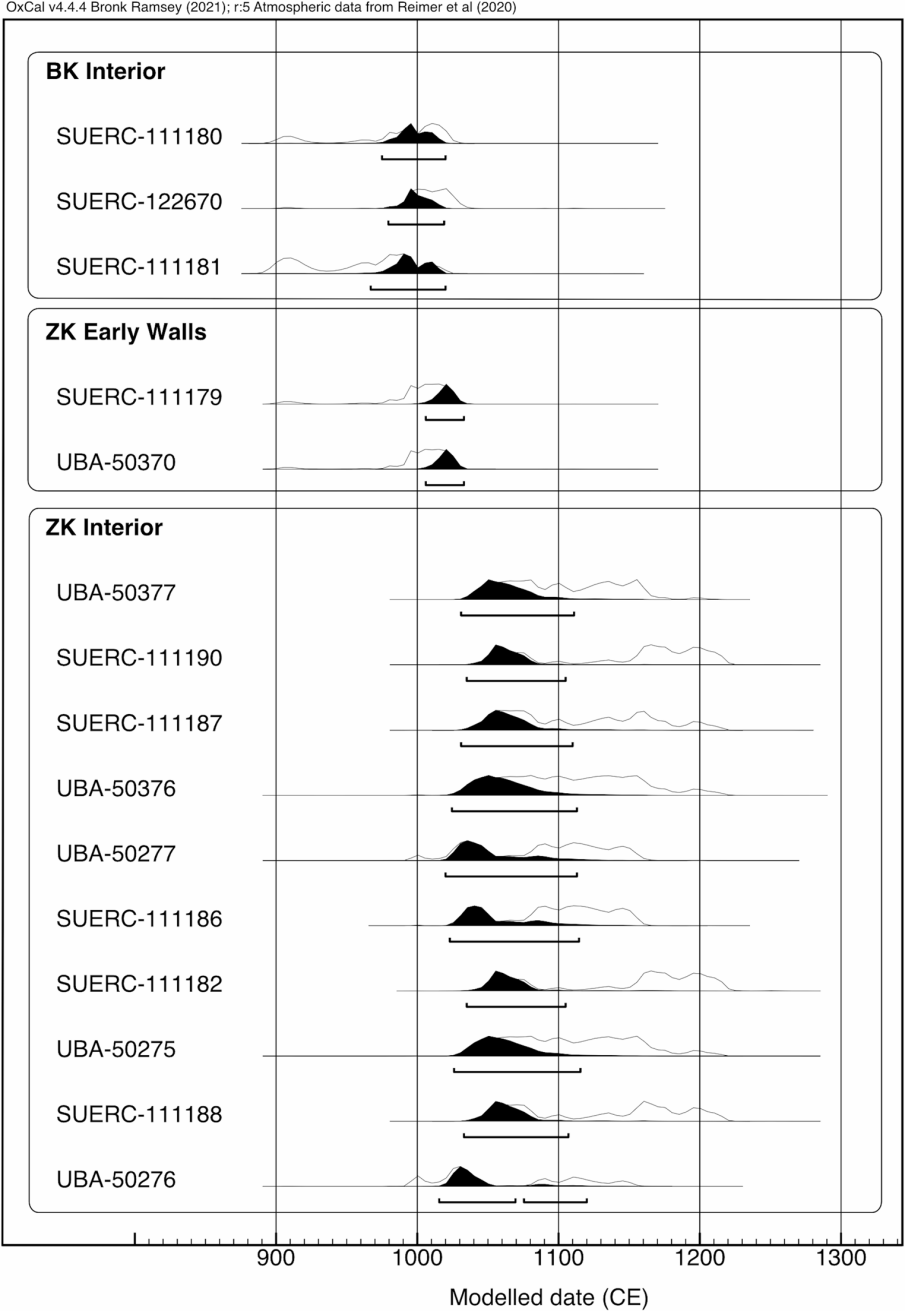
Modeled radiocarbon phases for the ZBK site based on fauna and charcoal samples from auger cores. Three phases are highlighted here, activity within the BK enclosure, deposits that were found near the original ground surface of the ZK walls, and activity within the ZK interior. Model produced using Oxcal online 4.4.4

Using 10 dates from the interior layers of ZK, excluding those used in the discussion of wall and ditch chronology above, the phase boundary tool of Oxcal 4.4.4 generated a modeled date range between 998 and 1027 CE to between 1052–and1164 CE for activity in the interior of ZK. The preponderance of probabilities in the model are in the mid 11th century CE (Fig. 9, table S3). There are two exceptions to this range (Table S3), the first in the sample at point 1652 (UBA-50278) that calibrates to the end of the 12th century. The second is 1530 (UBA-50372) that is older than the general range, spanning the 10th century CE. This is a carbonized piece of birchwood, the parsimonious explanation is that this was a burned fragment of an old log or beam.

Three dates from the interior of BK, two from bone below 30 cm in depth in widely separated auger cores, and one from a charcoal fragment at 125 cm depth in a large compound ditch provide a modeled date range of 941–1019 CE to 987–1023 CE for activity in the interior of BK. (Fig. 9, table S3).

Contrary to an earlier argument using historical sources the suggestion that ZK construction preceded BK by over half a century (Shiraishi 2008), the overall result of the radiocarbon dating of the interior of the two enclosures is that activity in BK predates that in ZK. However, the dates for the construction of the walls of ZK in the early 11th century CE are close to the period of likely midden formation in BK. The conclusion then is that the phasing of the sites, from currently available radiocarbon dates, is that BK was inhabited and walled around the same time as ZK’s walls were built, if not a short time before. The large compound in BK is likely the oldest such large structural group. In the century or more after the building of ZK’s wall, activity and midden formation continued there while in BK there was little or no midden creating activity. Activity in ZK likely reached into the early 12th century CE.

### Other chronological indicators

Two other chronological datasets are available. The first is coins and second is glazed ceramics. As noted above, the minting dates of the coins found in ZK fit within or before the full extent of the radiocarbon date range.

Glazed ceramics are datable through an assemblage approach. In Zhao Limeng’s (2019) detailed glazed porcelain based chronology of medieval cities in Inner Mongolia and Northeast China it is the white glazes that form the central element of the chronology. Periodization is developed from both variations in qualities, and likely origins, of those glazes and the addition of different colored glazes to the assemblages. For example, the middle Liao period is characterized by a relatively limited number of white glazed sherds. The late Liao period glazed wares are equally divided between white wares from northeastern kilns and finer quality white wares made in eastern central China. With the addition of small amounts of blue and white or darker green glazes. The Jin period (1115–1234 CE) saw an increase in fine white wares and yellow-brown glazes. Finally, the Yuan period (1271–1368 CE) included substantial proportions of blue to purple *jun* ware, light green celadon glazes, including examples from the Korean Peninsula, and decoratively painted black and brown on white *cizhou* ware (Zhao 2019, 43–54).

Following this typology, ZBK’s glazed ceramics have the yellowish fabric and tone of white that matches them with the Jin dynasty or Liao period Gangwayao kilns as well as a few finer white wares (Zhao 2019, Figs. 2, 3, 4 and 5). The majority of this glazed ware assemblage would fit into Zhao’s Late Liao category, with some tendency towards a Jin Dynasty assemblage. This, combined with a subset of the radiocarbon data, suggests continued habitation of ZK at least as Liao power waned and the material impact of the Jin state reached Mongolia.

## Discussion

The archaeological material we found at ZBK was not surprising. The ceramic forms, fauna, chronology and constructions were what we would expect from a site of the medieval era in Mongolia. The greatest value in this work was the full coverage, controlled and contextualized data that we collected.

### Methods

The methods used in this study were unfamiliar ones for archaeology in Mongolia. Augering has been used for the detection and definition of ephemeral sites (Gardner and Burentogtokh 2018), but not as a tool to examine and define urban sites. How effective was this approach? Our work demonstrates that it is a quick methodology that does not require a lot of artifact storage and, paired with photogrammetric mapping, can define an urban site’s archaeological elements.

Comparing the older excavations and augering is not straightforward, but broadly the more rapid methodology confirmed things such as the extent and depth of the sites, their chronology, the typical faunal assemblage, and the fabric of walls, roads, and structures. It did not provide the accuracy that excavation does in terms of construction details and subsistence data. However, the framework shown here was established with only c. 1% of the volume of excavation when compared to two seasons of digging.

There are some disadvantages of the auger methodology. The height of the auger bucket, 20 cm, and the grinding aspect of its use result in an inability to reliably detect compacted layers such as floors and even layers of rammed earth. Perlee’s excavations noted such layers and they are visible in section drawings, but none were detected in auger cores. The impact of augering action also damages osteological remains. Though we recovered a surprising amount of fauna, larger elements were broken in recovery making a full comparison with an excavated assemblage unreliable.

### Project objectives

Our work at ZBK aimed to examine three aspects of the site in particular, evidence for the nature of administration at the site, the contrast of empty and built up spaces within the walls, and the chronology of the enclosures.

#### Administration

In the research design for the ZBK project the definition of administrative structures was a simple one, prominence and tile roofs. Our systematic examinations recorded large compounds with tall tiled roofed structures and access to restricted spaces controlled by gates and walls. These could clearly have been elite residences or administrative working spaces. In BK, the south-facing possibly open fronted pillared building near the south central area of the site overlooked the Kherlen wetlands evokes elite compounds at other places along the Kherlen system and in the Gobi region (Rogers 2017; Shiraishi et al. 2024). Single isolated structures like this as well as the wide avenues of approach to them created a theater of power (Inomata and Coben 2006, Ueda et al. 2016), but a performance in a minor key was created by turning paths and restricted enclosures.

Where Baruun Kherem’s layout suggests an elite central place and its surrounding fief, ZK’s layout is dominated by several large compounds set back from the main roads. This suggests competing powers with their townhomes, perhaps connected to rural or mobile populations or other local aggrandizing positions.

#### Empty spaces

The ZBK project sought to examine the evidence for activity in the spaces inside the walled enclosures that appear to be empty. If these spaces were empty or used for short-term dwelling this speaks to the nature of these sites as permanent towns, fortified camps, or monumental central places, and to the experience of dwelling within the walls. At the outset it was clear that BK appeared to be mostly empty space, while ZK appeared to be occupied by structural remains and surface ceramics.

The primary data that were used to examine the empty spaces were first, cultural soil layers found in augering and ways in which they differ from the undisturbed soil column in the area. Second ceramics found in augers and on the surface. Sub-surface ceramics are indicative of the long term use of a space resulting in midden accumulation, structural decay, and perhaps disposal areas or pits. Ceramics found only on the surface suggest use in times of little soil accumulation or for short periods.

The northeast quadrant of ZK contained many fewer sub-surface artifacts and occurrences of fine grey layers were infrequent and where they were present they were thinner than elsewhere in the Zuun Kherem enclosure. Most cultural layers and ceramics were clustered around two groups of platform mounds visible here. The rest of the area shows unmodified soil and sand layers atop the typical reworked loess and gravel of the immediate area surrounding ZK. The conclusion is that there is no extensive buried cultural layer or other structures here, but that most likely this quadrant of the enclosure was empty.

In BK, there were few visible structures on the surface and auger cores produced a limited amount of pottery from any sub-surface deposits. Sediment layers follow a consistent pattern of fine brown surface soil above pale loess or reworked loess — the natural soil of the area and undisturbed by the deposition of cultural layers. The conclusion is that there was never impactful habitation in most of the BK enclosure. What surface ceramic assemblages there are here have the same composition of vessel types as those outside ZK’s walls, suggesting some typical mobile campsites that left no midden or structural soil behind. Generalizing to empty spaces within medieval enclosures, though animal penning is still a possibility pending geochemical studies, this evidence suggests these were empty spaces used for limited temporary habitation.

#### Chronology

Overall, the three dating approaches used here agree on the period of activity at ZBK, beginning in the early 11th century CE, leaving its densest archaeological signature during the middle 11th, and fading out after the early-12th century CE. This date range is not a surprise for a Kitan-Liao period city, fitting in with the beginning of Liao political focus on the Mongolian Steppe in 994 CE and the collapse of the Liao state in the mid-12th century. However, individual dates go well beyond the middle 11th century reduction in Liao state support for its Mongolian towns. Suggesting that Zuun Kherem at least had attained a place of permanence in the local social landscape and economy that may have allowed it to endure without extensive long-distance support.

The dates here suggest that the large compound in BK might be the earliest elite complex across the two enclosures, but secure dates from the ZK compounds are needed to confirm this. Because the cruciform avenue of BK does not match the alignment of gates in the walls, it is possible to see it as preceding the walls, whose symmetries would have determined gate locations. The chronological narrative at ZBK is then an emplacing of elite individuals into a powerfully structured space, the building of both town walls, and finally the inhabitation of ZK by people living in large and small compounds. Dated middens show intense deposition in ZK, but do not show any dates for early use or the construction of buildings there.

Our research lays the groundwork for three major avenues of future research at Zuun Barun Kherem. The first, and most obvious, in excavation of structures and kilns to provide detailed narratives of inhabitation and power within the sites. The second is a soil chemistry study to complete the work studying the function of empty spaces with enclosures. And third, to compare ceramic assemblages, and other material, to the non-urban areas of Kitan-Liao period settlement found in regional surveys to examine the relationship between urban and non-urban habitation.

### Was inhabitation during the Kitan century in Mongolia sustainable?

The 1344 CE Liao Shi records in the biography of the minister Xiao Hanjianu that the Kitan imperial court debated the sustainability of their frontiers in Mongolia, with Xiao Hanjianu arguing that the Liao should withdraw from the Orkhon and Tuul river basins in the central Mongolia plateau and strengthen their frontier further east in the Kherlen (Toqto’a 2016: 103.1594-1598; see also Wittfogel and Feng 1949: 557–559). In this account, we glimpse a part of this debate in which the difficulties in provisioning and defending the sites almost two generations after they were first populated were highlighted. Today, we are still faced with this question. How self-sustaining were the settlements in the Kherlen valley? Archaeological examination of Zuun Baruun Kherem by the ZBK Project, and earlier excavations by Perlee, suggest that the inhabitation of established towns and communities might have been sustained for generations after the fading of imperial support. We see here glimpses of a system of herding, farming, fuel management, and ceramic production, all sheltered by strong walls.

Our limited findings from ZBK have allowed us to draw some outsized conclusions about living within the site. The built areas of the towns were dominated by high, tile roofed buildings, some with decorated roof-combs. The spaces around these were sheltered, divided into many small enclosures and courtyards contrasted with wide open ground and wide avenues. Perhaps, like a modern Mongolian small town, people went about the big spaces on horseback. During the long cold of winter the sheltering walls would have been welcome. Rhythms of life included bringing in wood fuel from some distance away, taking sheep and goats in and out of the town, the smoke from kilns carried away southward, and the clay to make ceramics and tiles dug from the many river banks nearby.

The earthen fortification walls with their many bastions and protected gates presented a stern external face, with rain the ditches around them filled with water and they rapidly become marshy ground. The state-of-the-art military architecture of both enclosures (Pursey, Wright et al. in prep.) suggests the presence of soldiers or militia in the populations who might rush to the walls in times of crisis. Though the lack of military detritus in the sites, argues against an extensive garrison. We can guess at the presence of long term residents and families, as well as the elite and their entourages. With the *girs* of shorter term residents setup in open ground within the walls, and close by them.

The lay of the land around the enclosures, with Baruun Kherem slightly higher than and sloped towards Zuun Kherem and lower ground in between, would have made the walls easily intervisible, though not from within the walls of Zuun Kherem. At around 2 km apart, moving people and animals at the gates or on the walls would have been visible without much detail and sound crossing the distance only in the calmest air. What this proximity would always allowed, however, was the reminder of the contrast between the sites, the elite business of Baruun Kherem, contrasted with the quotidian life and management of Zuun Kherem and the contrast of a center focused imperial outpost with a frontier colony.

## Supplementary Information

Below is the link to the electronic supplementary material.

Supplementary figure 1 (PNG 999 KB)

(TIF 16.2 MB)

Supplementary figure 2 (PNG 1.06 MB)

(TIF 19.7 MB)

Supplementary figure 3 (PNG 1.02 MB)

(TIF 16.4 MB)

Supplementary figure 4 (PNG 1.11 MB)

(TIF 19.8 MB)

Supplementary figure 5 (XLSX 23.2 KB)

Supplementary figure 6 (XLSX 23.4 KB)

Supplementary figure 7 (XLSX 19.3 KB)

## Data Availability

All data, digital and collected artifacts, are archived with the Archaeology Center of the National University of Mongolia. Upon completion of the publication and other work related to the project the digital data (auger cores, artifact counts, and GIS layers) will be archived with Archaeological Data Services (ADS) https://archaeologydataservice.ac.uk.
