# Supplementary figures and images for: Zuun Baruun Kherem, a medieval Eurasian center in Eastern Mongolia
Source: Asian Archaeol. 2025 Oct 7;9(2):175–96. doi: 10.1007/s41826-025-00113-2 (PMC12799725; doi:10.1007/s41826-025-00113-2)

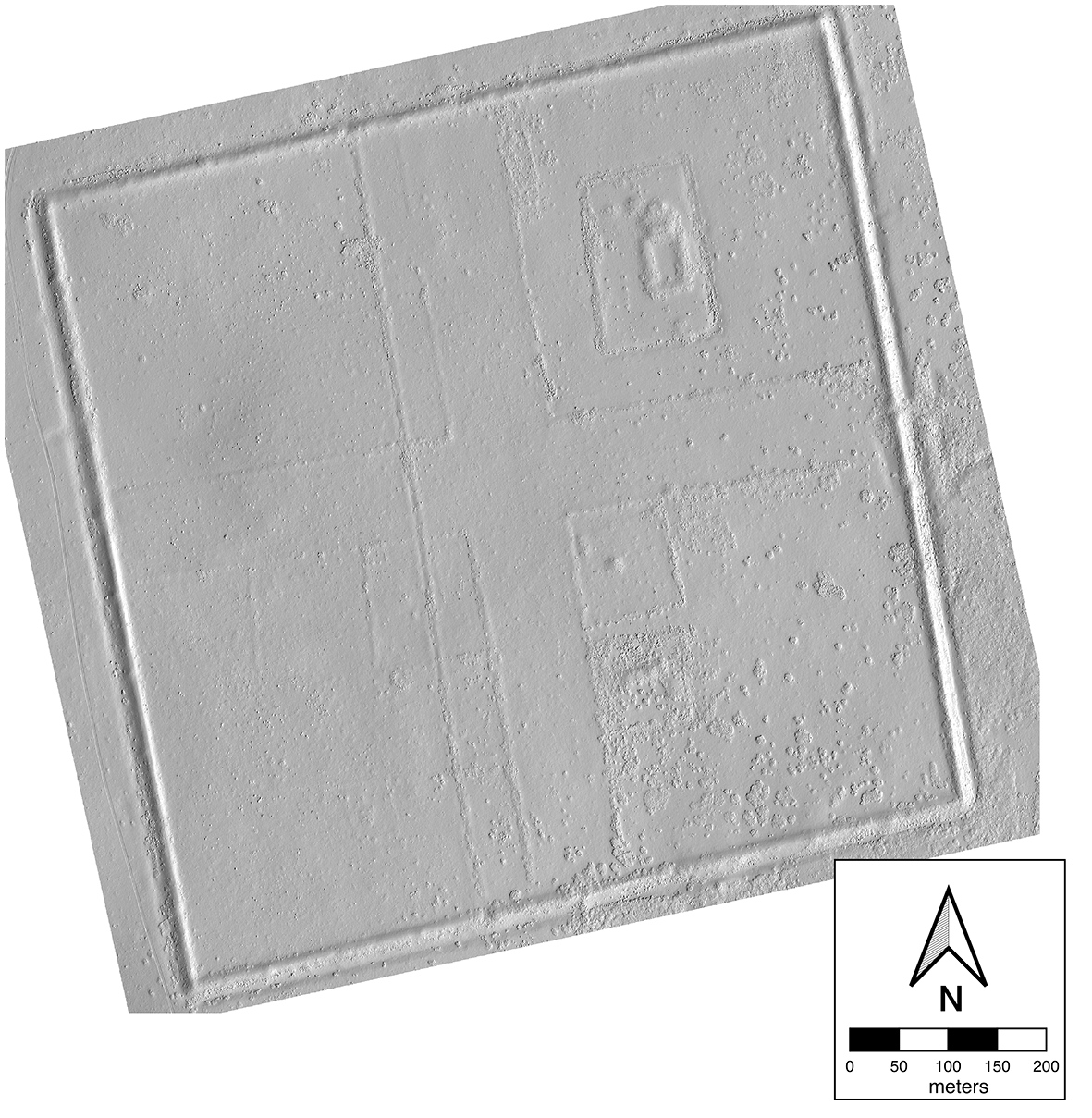

Supplement: Supplementary file 1 — (PNG 999 KB) [file 41826_2025_113_Fig10_ESM.png]

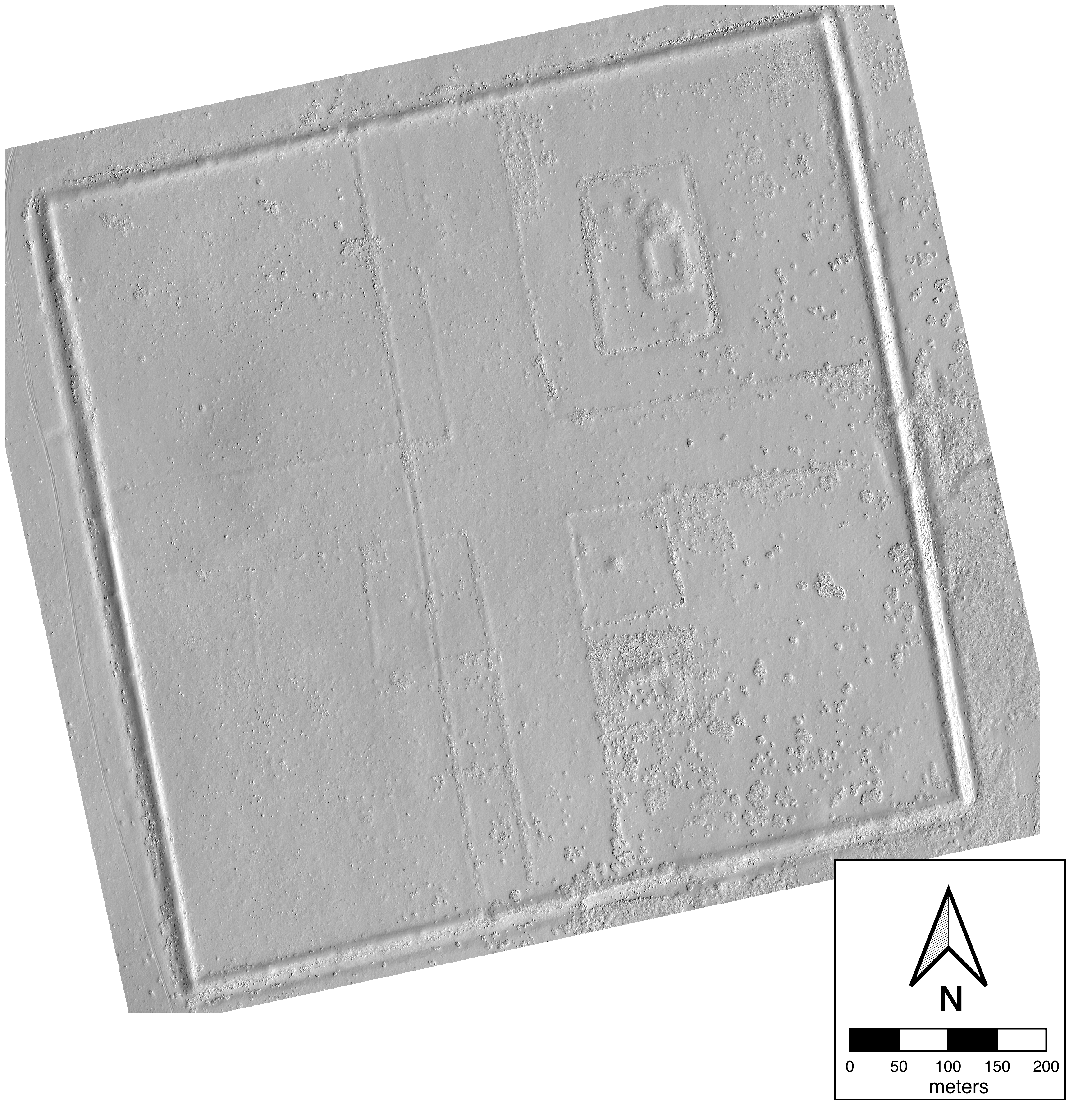

Supplement: Supplementary file 2 — (TIF 16.2 MB) [file 41826_2025_113_MOESM1_ESM.tif]

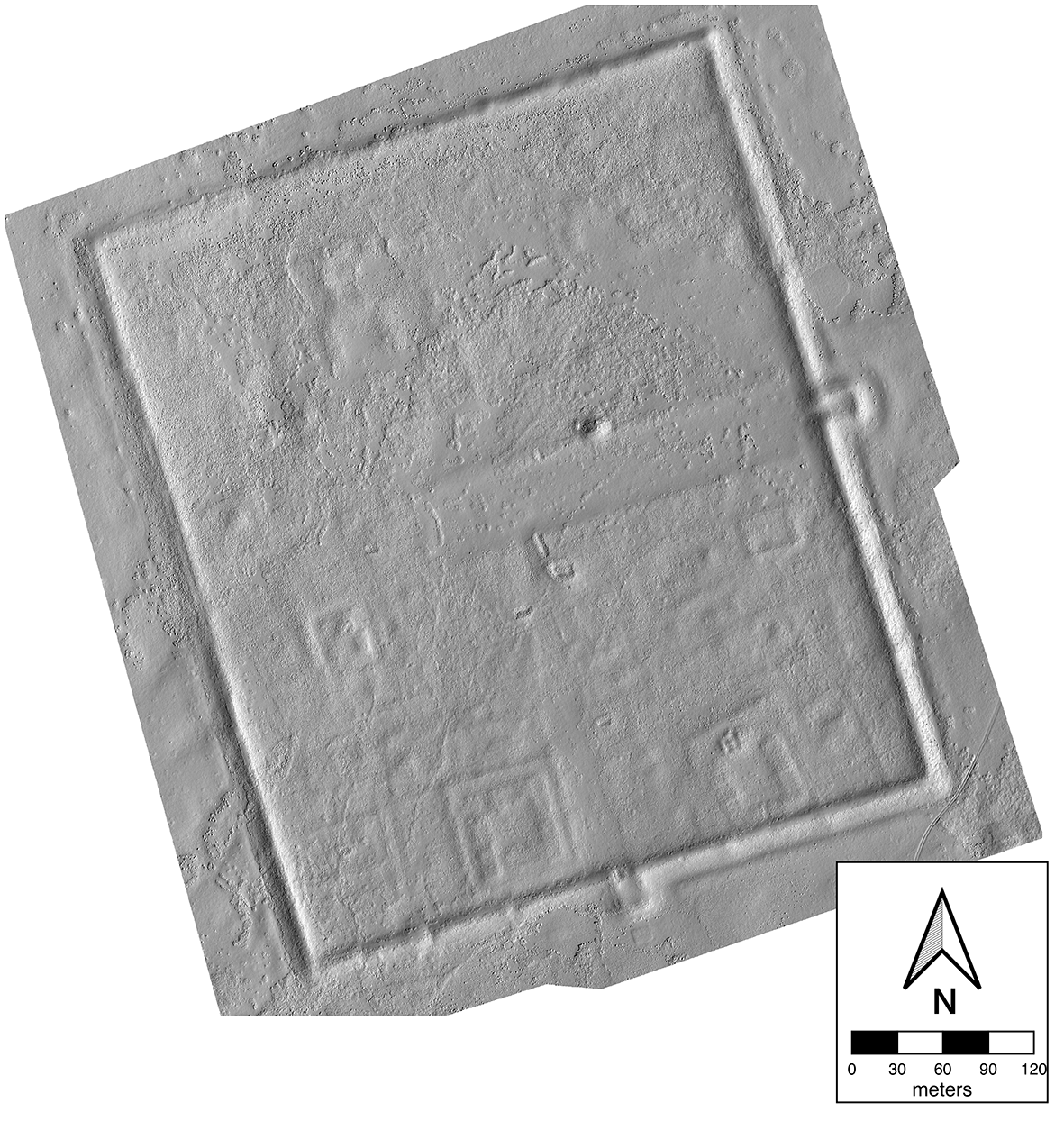

Supplement: Supplementary file 3 — (PNG 1.06 MB) [file 41826_2025_113_Fig11_ESM.png]

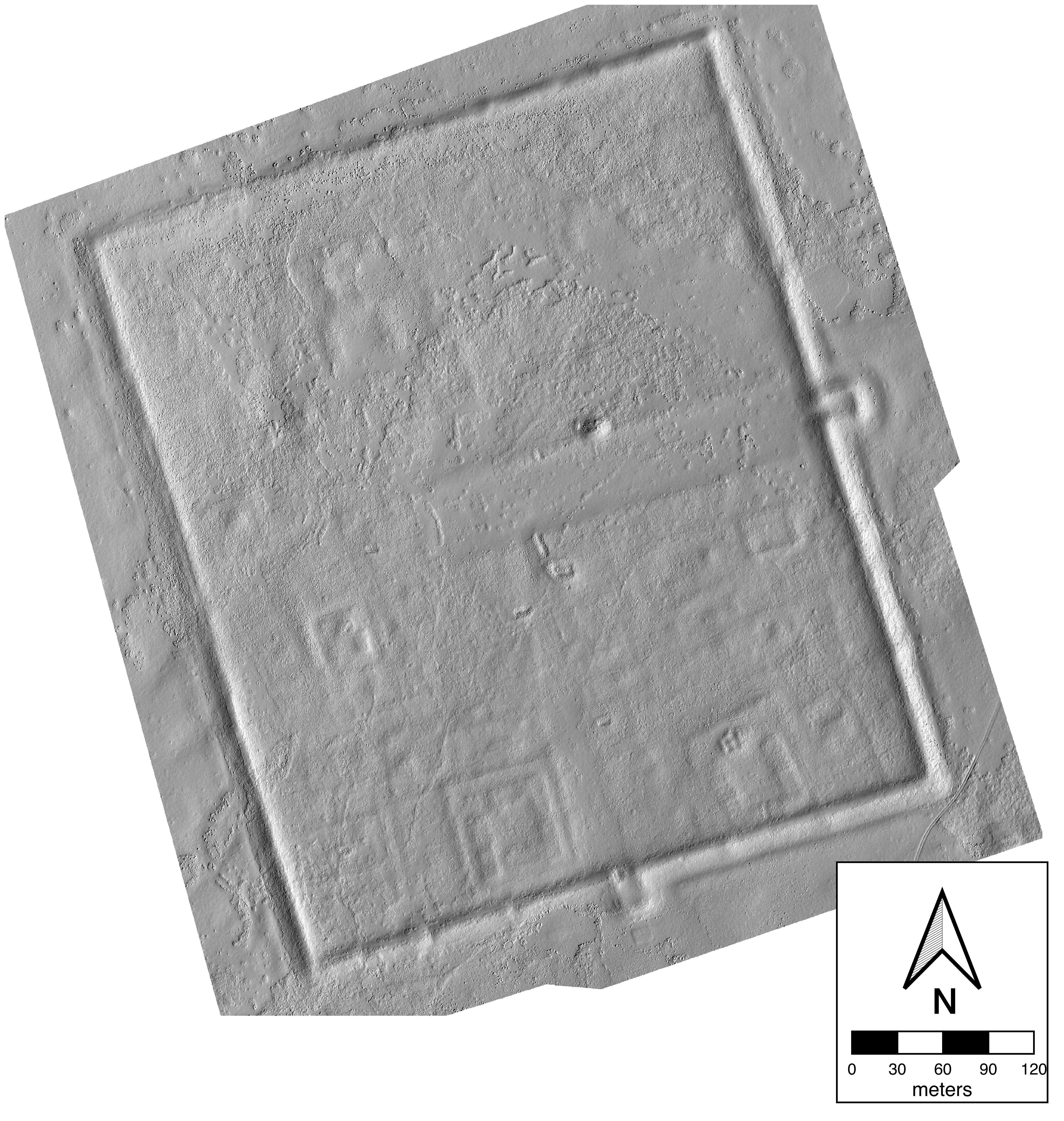

Supplement: Supplementary file 4 — (TIF 19.7 MB) [file 41826_2025_113_MOESM2_ESM.tif]

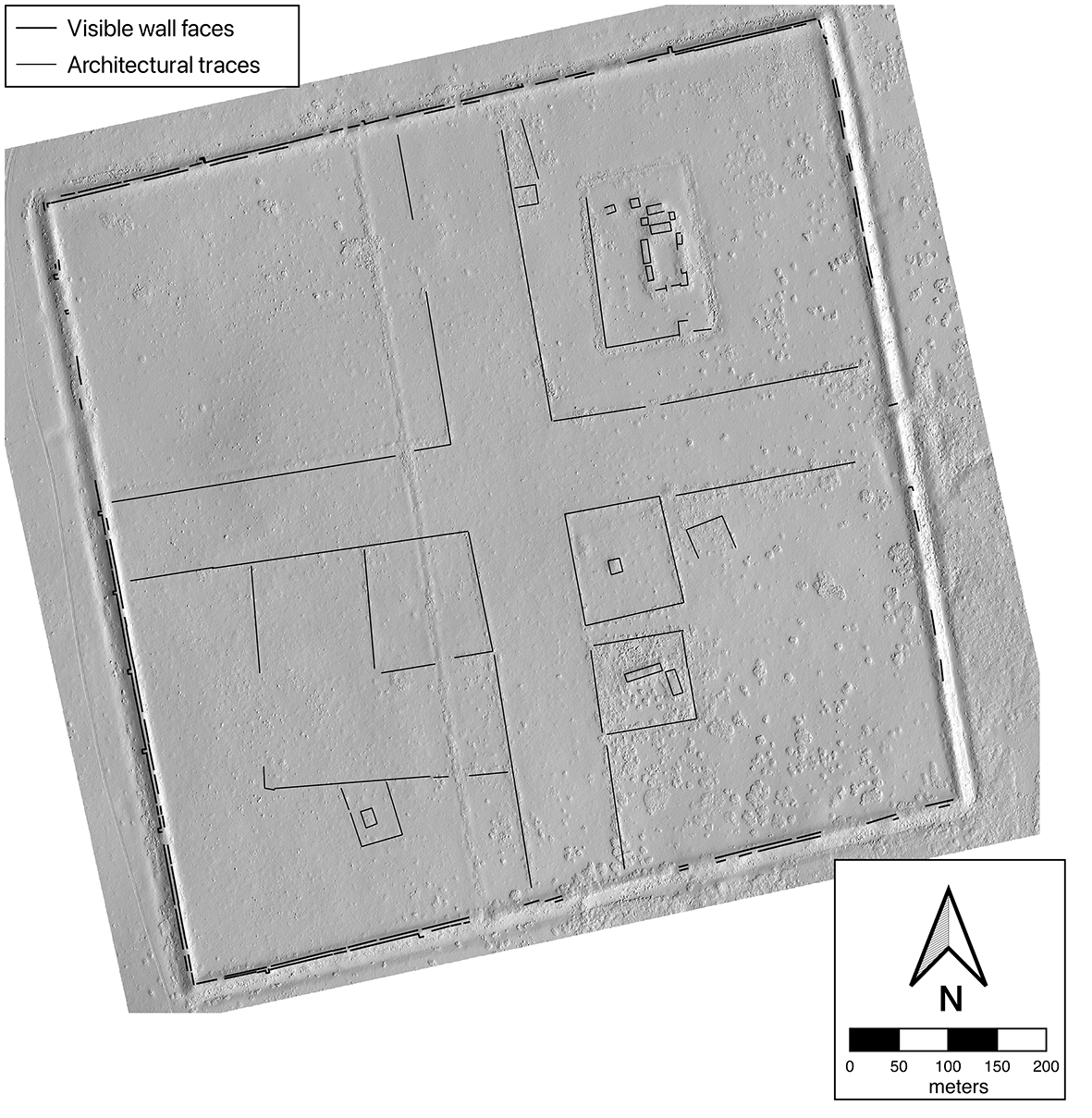

Supplement: Supplementary file 5 — (PNG 1.02 MB) [file 41826_2025_113_Fig12_ESM.png]

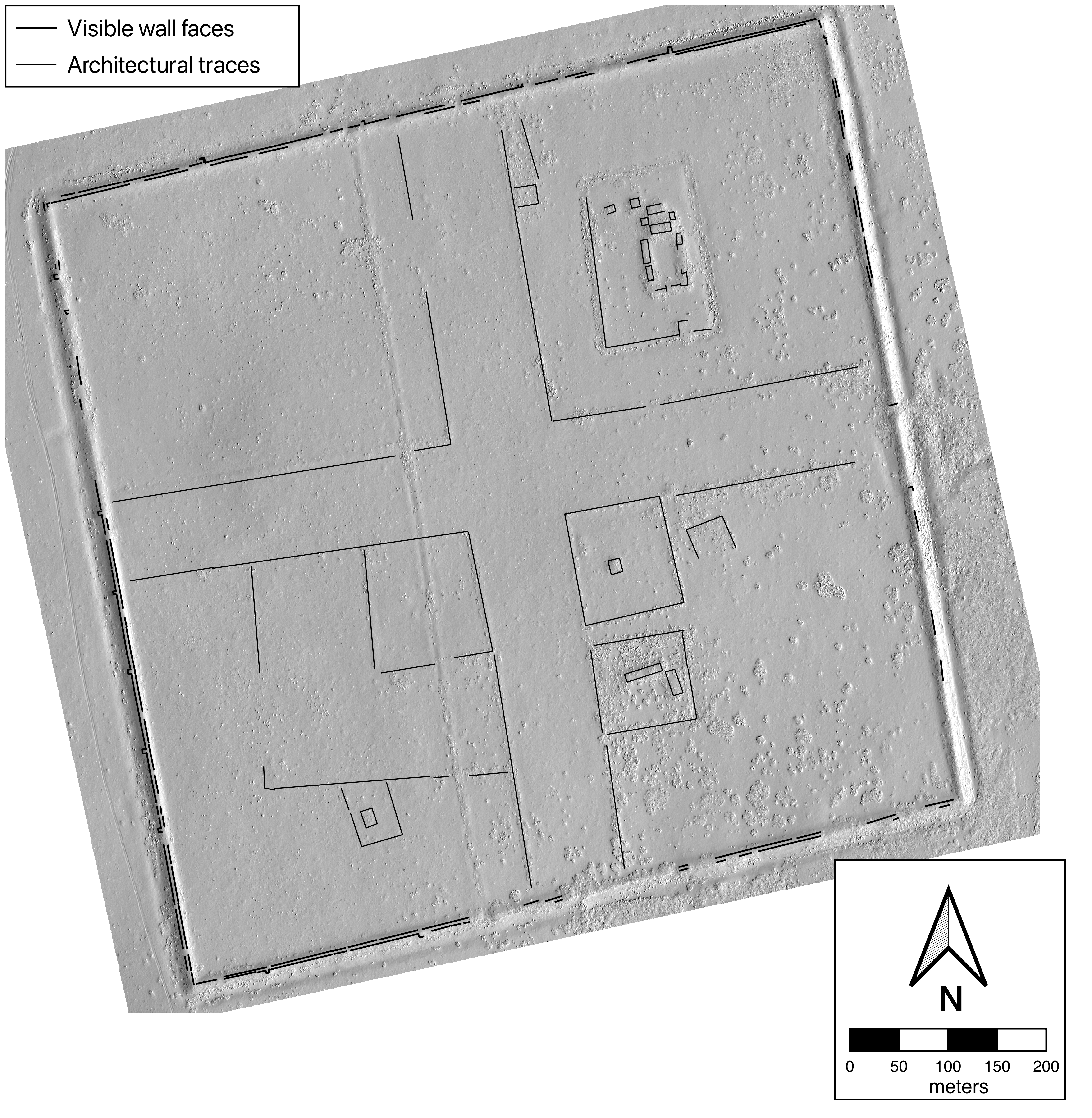

Supplement: Supplementary file 6 — (TIF 16.4 MB) [file 41826_2025_113_MOESM3_ESM.tif]

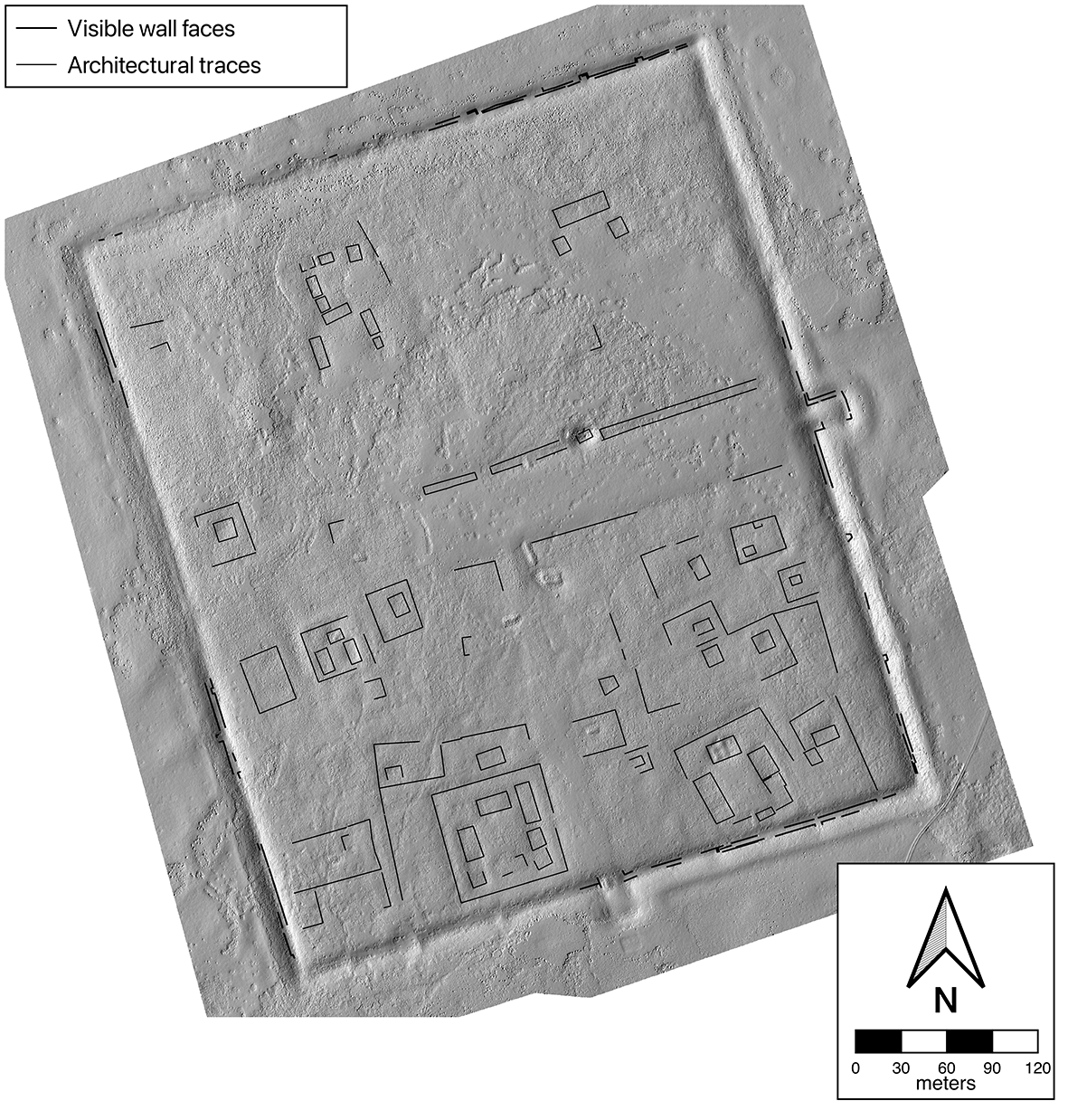

Supplement: Supplementary file 7 — (PNG 1.11 MB) [file 41826_2025_113_Fig13_ESM.png]

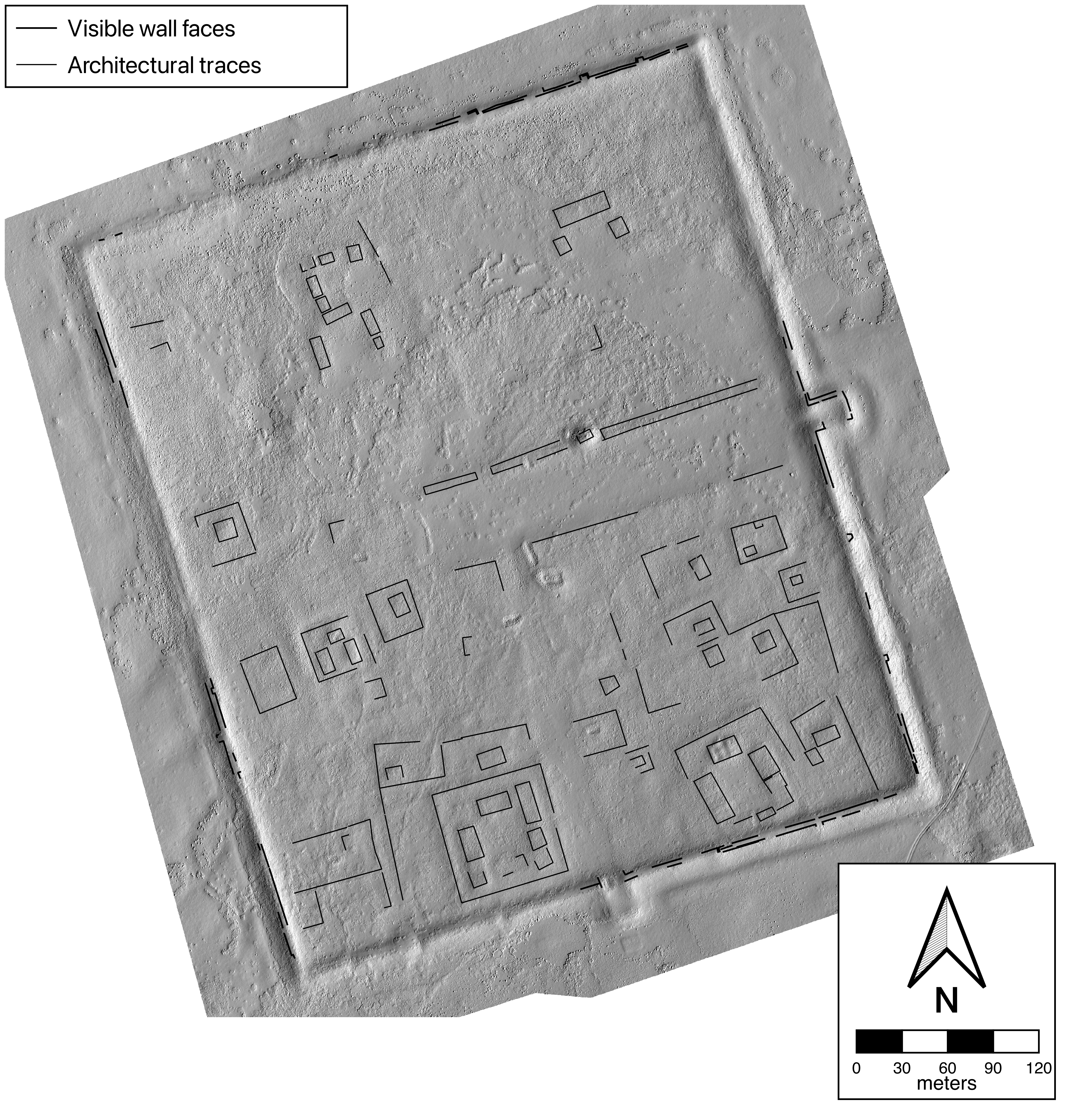

Supplement: Supplementary file 8 — (TIF 19.8 MB) [file 41826_2025_113_MOESM4_ESM.tif]
